# Investigating the Goldilocks Hypothesis: The Non-Linear Impact of Positive Trait Change on Well-Being

**DOI:** 10.1371/journal.pone.0131316

**Published:** 2015-07-10

**Authors:** Chris C. Martin, Corey L. M. Keyes

**Affiliations:** Emory University, Atlanta, Georgia, United States of America; University of Vienna, AUSTRIA

## Abstract

This paper attempts to reconcile two perspectives on the impact of positive trait change. The first perspective views positive trait change as salubrious because it reflects the process of self-enhancement, whereas the second perspective views positive change as costly because it disrupts the self-verification process. We propose that benefits and costs accrue at discrete rates, such that moderate positive trait change is more beneficial than too little and too much positive change. This constitutes a Goldilocks hypothesis. Using the MIDUS longitudinal dataset (N = 1,725) we test this hypothesis on four traits, namely, social extraversion, agentic extraversion (agency), conscientiousness, and neuroticism. The Goldilocks hypothesis was supported for social extraversion, agentic extraversion (agency), and conscientiousness. Reduction in neuroticism seemed uniformly predictive of higher well-being. Thus, not all amounts of positive trait change are beneficial. While we find no evidence for a limit to the benefits of reduced neuroticism, there is a “just right” amount of positive change in extraversion and conscientiousness that results in the highest level of well-being. Our findings suggest that non-monotonic models may be more valid in investigations of personality change and well-being.

## Introduction

Personality traits change throughout the lifespan [1]. There are differing views on the costs and benefits of this change. Some psychologists have marshaled evidence to support the claim that stability lies at the root of greater well-being, and they portray both growth and decline as costly to well-being [2]. Others have demonstrated that positive trait change causes greater well-being, and suggested that growth, no matter how much, is beneficial [3].

We investigate the merits of both perspectives, reconciling them in a way that reflects the Goldilocks hypothesis. That is, when it comes to subjective well-being (SWB), a small amount of positive trait change isn’t enough, and a large amount of positive trait change is too much, but a moderate amount of change is conducive to promoting well-being. We investigate the Goldilocks hypothesis of positive trait change using non-linear modeling of the impact of trait change, because the Goldilocks hypothesis predicts a non-monotonic relationship of positive trait change with subjective well-being (SWB). Over time, SWB should increase linearly with positive trait change until it reaches an apex after which continued change results in decreasing SWB.

### Perspectives on Stability and Change

There is a dialectical opposition between continuity and change in classical thought. In support of change, Socrates uttered his famous dictum, “Know thyself,” thus idealizing the continuous accumulation of self-knowledge across one’s lifespan. In contrast, the Stoic philosophers put forth a moral vision of statis and indifference. To attain the ideal life, Stoics argued, individuals should learn to accept the vicissitudes of life and remain unperturbed by them. For Stoics, personality stability rather than growth was ideal. Similarly, the philosophical traditions of East and South Asia, which include Hinduism and Buddhism, emphasize equanimity, a form of personality stability. In such traditions, a common feature is the sacralization of a liberated state (e.g., *nirvana* in Buddhism, or *moksha* in Hinduism), after which human striving ceases and stability is attained.

Today, one sees a greater emphasis on change and growth, especially in Western cultures, a phenomenon that derives from the legacy of existentialism and humanism. Existentialist philosophers emphasized struggle, personal transformation and distinctiveness as being necessary to an authentic life. Humanists like Carl Rogers claimed that psychological health resulted from becoming one’s “true” or authentic self [4]. In short, positive personal change was framed as beneficial and necessary to an individual’s well-being or happiness. Such framing is evident in synonyms of positive change, which include *self-enhancement*, *improvement*, *growth*, *attainment*, and *maturity*.

### Personality Trait Change Over the Lifespan

Trait profiles have high rank-order stability, indicating that a person’s characteristic traits—those that are markedly high or low in strength—remain persistently high or low relative to the person’s other traits [5]. However, research on absolute levels of traits shows that gradual change occurs as individuals age [6,7]. Conscientiousness and the social-dominance facet of extraversion increase, while neuroticism decreases, from young adulthood onward [7]. The social vitality facet of extraversion is stable in middle adulthood but declines in old age, and openness to experience evinces a curvilinear (inverted-U) pattern of change. These findings of personality trait change in adulthood have led to newer research on the costs and benefits of trait change.

### Arguments for the Benefits of Positive Trait Change

Arguments in favor of trait change are predicated upon the meta-analytic evidence regarding the association between traits and SWB. People with higher levels of extraversion and conscientiousness, and a lower level of neuroticism tend to have higher well-being [8]. Given these relationships between trait levels and well-being, more positive trait change is presumed to lead to greater well-being.

Personality psychologists have posited two mechanisms that connect traits to well-being: instrumental and temperamental [9,10]. According to the instrumental view, people who have high levels of adaptive traits act upon their environment in ways that promote their own happiness. Instrumental behaviors increase the likelihood of experiences that increase well-being, and decrease the likelihood of experiences that cause stress or deprivation. According to the temperamental view, traits indicate the configuration of a person’s psychological system, which affects well-being. The temperamental theory that has received the greatest support is reinforcement sensitivity theory [11], which specifically relates to two traits: extraversion and neuroticism. According to this theory, there are three systems within the human brain to deal with punishment and reward. The behavioral activation system (BAS) motivates appetitive behavior to satisfy one’s needs. The fight–flight–freeze system (FFFS) motivates aversive behavior to escape from threatening situations. The behavioral inhibition system (BIS) manages situational ambiguity and decisional uncertainty. Extraversion is linked to greater sensitivity in the BAS; neuroticism is linked to greater sensitivity in the BIS and FFFS. As a result, people who are very extraverted tend to be zestful and approach-oriented, whereas people who are very neurotic tend to be nervous and avoidance-oriented. Other variants of instrumental theories and structural theories can also be found in the literature [12].

Another avenue of research links traits with well-being through differential motivational habits. The implicit motives tradition posits that people are unconsciously driven to satiate specific needs, and traits represent styles of goal pursuit [13]. For instance, differences in extraversion influence styles of affiliative pursuit. Individuals high in extraversion are more likely to pursue affiliation directly through in-person social gatherings, while those low in extraversion may pursue affiliation indirectly through letter writing or e-mails. Because some styles of goal pursuit lead to greater satisfaction than others (e.g., high extraversion leading to direct affiliation), people who adopt a relatively efficacious style experience greater satisfaction. We turn next to the literature showing the benefits of three particular traits in terms of higher levels of extraversion and conscientiousness, and lower levels of neuroticism.

Agreeableness and openness to experiences are also associated with well-being, but their relationships tend to be moderated by factors such as life events. In addition, their associations tend to be weaker in magnitude, relative to the traits examined here [14,15].

#### Extraversion

Extraverted people are happier than introverted people [16,17], a finding that been robustly replicated across cultures [18]. Because extraversion reflects the tendency to experience positive affect [19], it appears to buffer against depression [20–22]. When instructed in an experimental setting to behave extravertedly, even introverted people experience greater positive affect [23].

High levels of extraversion represent high appetitive reactivity [24] or a highly sensitive approach system [25]. These are appetitive systems that drive the pursuit of rewarding outcomes, rather than the avoidance of painful outcomes. People who have a strong behavioral activation system are therefore more sensitive to opportunities for positive outcomes. Highly extraverted people want affective rewards more strongly than less extraverted people [26], and also maintain a more positive baseline [27]. In short, with increasing extraversion, individuals are doubly advantaged—they have a higher level of positive affect than the average person, and their positive affective system is greedier.

#### Conscientiousness

Conscientiousness is the tendency to control one’s impulses, follow social norms of order, set goals, and prefer orderliness [28]. Conscientiousness is positively associated with positive affect, happiness, and life satisfaction. It correlates with SWB at levels comparable with extraversion and neuroticism [29–31]. Conscientiousness predicts success in telic pursuits [32]; greater occupational attainment and earnings after cognitive ability is controlled for [33]; better self-care of one’s health and less addiction [34]; and lower risk for chronic and acute illnesses [35,36]. Conscientiousness also predicts many behaviors conducive to better health. Thus, conscientiousness may engender well-being through its promotion of successful telic pursuits, occupational advancement, and healthy behaviors.

#### Neuroticism

Neuroticism, which is the tendency to experience strong and enduring negative reactions to stimuli, is one of the most robust predictors of negative affect (NA) and psychopathology [37]. Highly neurotic individuals are less likely to be affectively moved by positive stimuli than extraverted people [38], and high levels of neuroticism indicate a strong avoidance system [39], or jointly strong behavioral-inhibition and fight–flight–freeze systems [11]. Greater attention is paid to painful outcomes, and greater effort exerted to manage such outcomes. When neurotic people feel that they are in ill health, for example, they complain of more symptoms and are more likely to seek treatment [40].

Neuroticism is associated with lower job satisfaction [41], lower self-efficacy and goal setting [42], and a host of negative life outcomes [43,44]. It is also associated with a variety of risky and unhealthy behaviors, including smoking [45], alcohol abuse [46], unprotected sex [47], and criminal arrest [48]. Moreover, neuroticism predicts the earlier onset of chronic illnesses [49] and depression [50].

### Arguments for the Costs of Positive Trait Change

Positive trait change may lead to perceptions of improvement, but it may also lead to perceptions of self inconsistency. The self-system theory (SST) focuses on the paradoxical impact of such perceptions, which constitute temporal comparisons of perceived improvement relative to stability [51]. People use temporal self-comparisons to ascertain personal change or continuity. They examine the similarity of some facet of the self in relation to the past and, in turn, they indicate how much they believe they have changed or stayed the same. Judgments about change, couched in evaluative terms such as *better* or *worse*, reveal whether people feel that their personal qualities, abilities, functioning, or aspects of life have improved or declined [52,53].

According to SST, the standards of self-consistency and self-enhancement are germane to predicting whether people will view positive trait change (i.e., improvement) as good, bad, or both. Consistency is the principle of viewing oneself as the same over time, while enhancement is the principle of seeing oneself as better than others or, as is relevant to this study, as a better person over time [54]. Individuals use both self-standards to understand themselves, judge themselves, and self-regulate their behavior. Individuals desire feedback that is consistent with and flattering of the self [54–60]. Self-consistency and self-enhancement provide individuals with confidence—a sense that their life is predictable—and self-enhancement also provides individuals with the sense of overall worth [56,61–63].

People react and respond to judgments of their experiences through the channels of affect and cognition [64,65]. When information or experience can be judged as uniformly bad or good, individuals’ feelings and thoughts about that experience are aligned. Good experiences lead to positive feelings and positive thoughts; bad experiences lead to negative feelings and negative thoughts. When experience and judgments are a mixture of good and bad, feelings and thoughts can be misaligned, triggering cognitive-affective crossfire [66]. For instance, positive feedback to individuals with high or low self-esteem results in about equal levels of positive affect because the feedback satisfies the standards of self-enhancement. However, compared to those with high self-esteem, individuals with low self-esteem are less confident in the veracity of the positive feedback, because it violates the standard of consistency for those with low esteem [66].

Whereas Swann and colleagues [66] showed that some kinds of experience (i.e., receiving positive feedback) for certain kinds of people (i.e., those with low self-esteem) can generate divergent affective and cognitive reactions, the self-system theory (SST) extends this logic to the consequences of subjective change [51]. Perceived decline is uniformly bad because it violates the needs for self-consistency and self-enhancement. Stability satisfies the need for consistency and is neutral with regard to enhancement (i.e., it does not activate the standard of enhancement). However, perceived improvement satisfies the need for enhancement but violates the need for consistency. As such, SST predicts that perceived improvement will not result in better well-being, and will sometimes result in worse well-being than stability.

The SST of change has been investigated in three separate studies, each employing large, nationally representative samples of adults. Two studies focused on U.S. adults, and featured temporal comparison of changes in one’s functioning in social roles (spouse, worker, and parent) and in domains of life (e.g., work, intimacy, health) [51,67]. Perceived changes were linked to emotional well-being and psychological well-being. Data for study 1 came from the Midlife in the United States (MIDUS) study and sample, in which respondents completed scales of positive and negative affect. Respondents also evaluated their current and past (10 years ago) functioning as spouses (or close relationship partners), in employment, and as parents. As predicted, levels of positive affect decreased as the amount of perceived improvement and perceived decline increased; adults who saw themselves remaining the same had lower negative and higher positive affect than those who perceived themselves improving or declining.

Study 2 extended this research by investigating different measures of subjective well-being and different measures of subjective change in a nationally representative sample of over 1,000 U.S. adults. Functioning was assessed in the domains of health, finances, close relationships, work, physical appearance, and sexual functioning. Respondents evaluated their current functioning in each domain and whether and how much they saw their functioning in each domain as the same, worse, or better today than five years ago. The subjective well-being outcomes included a measure of dysphoria, a single item measure of life satisfaction, and Ryff’s [68] scales of self-acceptance and personal growth. The latter measures were included to validate whether perceived improvement is associated with a sense of growth even while it violates consistency and reduces acceptance of the self.

A key finding in study 2 was that adults who perceived more improvement over the six domains reported more personal growth than adults who remained the same; adults who declined reported less personal growth than those who remained the same. Nonetheless, levels of dysphoria increased, and levels of self-acceptance decreased, as the amount of perceived improvement (and decline) increased. The only exception was that adults who perceived more improvement reported no less satisfaction than those who remained the same. Whereas life satisfaction decreased in proportion to perceived declines in functioning, life satisfaction was orthogonal to perceived improvements. Thus, when compared with remaining the same, perceived improvement in life domain functioning was associated with more personal growth, but it was simultaneously associated with elevated dysphoric affect and less self-acceptance.

Study 3 [69] investigated perceived change and stability in former East and West Germans after reunification. Respondents evaluated how they perceived themselves currently (post-reunification) relative to ten years before (pre-reunification) in six domains. The six domains included spousal (or partner) relations, family relations, friendship relations, work, standard of living, and housing. Adults who reported more improvement had higher levels of negative affect and the same level of satisfaction and positive affect as those who perceived stability. Adults who perceived more decline reported more negative and less positive affect and less life satisfaction than adults who perceived stability.

Admittedly, the set of respondents who reported themselves as stable comprised two subsets: people who truly perceived themselves as stable, and people who were unwilling to report anything less than a positive self in order to seem socially desirable. Controlling for social desirability would have been ideal. Nevertheless, people who suppressed negative responses may have reported neutral-to-positive changes and positive-to-neutral changes, so such suppression does not entail ostensible stability.

### Reconciling the Perspectives: The ‘Goldilocks’ Hypothesis

Both sides have put forth valid arguments about change. Reconciliation requires the examination of a non-linear effect wherein the most beneficial outcomes are attained when the amount of positive change is neither too large nor too small. Thus, we posit an inverted-U effect. Grant and Schwartz [70] noted that many character strengths evince an inverted-U effect, such that a moderate level of the character strength predicts greater well-being (see also [71,72]). They found that this assertion held for numerous virtues: courage, humanity, love, justice, optimism, self-efficacy, and self-esteem. In some cases, the reason for dysfunctionality at a high level was quite straightforward—after crossing a certain threshold, a strength changes into a liability. Courage turns into foolhardiness, and optimism turns into overconfidence.

In other cases, the inverted-U effect represents the difference between positive and negative consequences: “good things satiate and bad things escalate” [73]. For instance, becoming more extraverted will incrementally satisfy the need for affiliation, and this will only extract a small cost in resources. However, once an inflection point has been crossed, a person will only incur greater costs, causing aggregate satisfaction to decline.

Positive trait change endows people with certain benefits—healthier life choices, less rumination, more approach-goal setting, and more sociality. At the same time, positive trait change extracts certain costs—discontinuity to the self through violation of self-verification. We posit that the benefits and costs accrue at distinct non-linear rates. As a result, trait change has a non-monotonic association with well-being. Compared to high amounts of positive trait change or stability, moderate change is optimal.

Because *absolute* trait levels predict well-being, we also expect to find a difference between people who have a stable high level of a trait and people who have a stable low level of a trait. If a trait is beneficial, people who have a stable high level of that trait will be happier than those who have a stable low level. The effect of stability itself will cancel out, given that both groups are stable, and thus trait level will be solely consequential.

In sum, we examine two hypotheses in this paper:

Hypothesis 1: Moderate growth in a beneficial personality trait (and moderate decline in a detrimental trait) will predict the highest level of individual well-being. Well-being scores will increase up to the level of moderate growth and then decline after crossing this point.
Hypothesis 2: Positive stability (i.e., the maintenance of a high level of a positive trait) will cause individuals to experience greater well-being than negative stability (i.e., the maintenance of a low level of a positive trait).

### Testing Hypotheses with Response Surface Analysis

A recent paper using the Midlife in the United States (MIDUS) dataset and the Big Five focused on the consequences of personality change on health and well-being outcomes [2]. This paper did not explicitly rely on the self-systems framework, but it rested on a similar theoretical foundation, namely, that a coherent sense of self is a psychological coping resource that buffers against stressful life events [74] and indicates greater agency or mastery [75,76]. Negative trait change across a ten-year interval in the MIDUS sample predicted a lower level on total psychological well-being, worse self-reported global health, and an increase in metabolic syndrome.

In the current study, we focus on the same predictors in the same MIDUS dataset but we rely on a quadratic model. Moreover, we do not use algebraic and absolute difference scores. Although difference scores can be useful in panel studies with exactly two waves, they also have weaknesses, the most pertinent being that difference-score models assume linear relationships [77,78]. Difference scores are justifiable only if one finds that no additional variance is explained after adding quadratic and cubic terms.

Problematically, Human and colleagues [2] also aggregated change across distinctive traits in order to derive an indicator of personality change. Aggregation glosses over the distribution of change across traits. In aggregated analyses, a person who changes by 10 points in one trait gets the same change score as a person who changes 2 points in each of five traits. Thus, aggregate scores can be misleading when one’s dependent variable is well-being.

**Fig 1:**
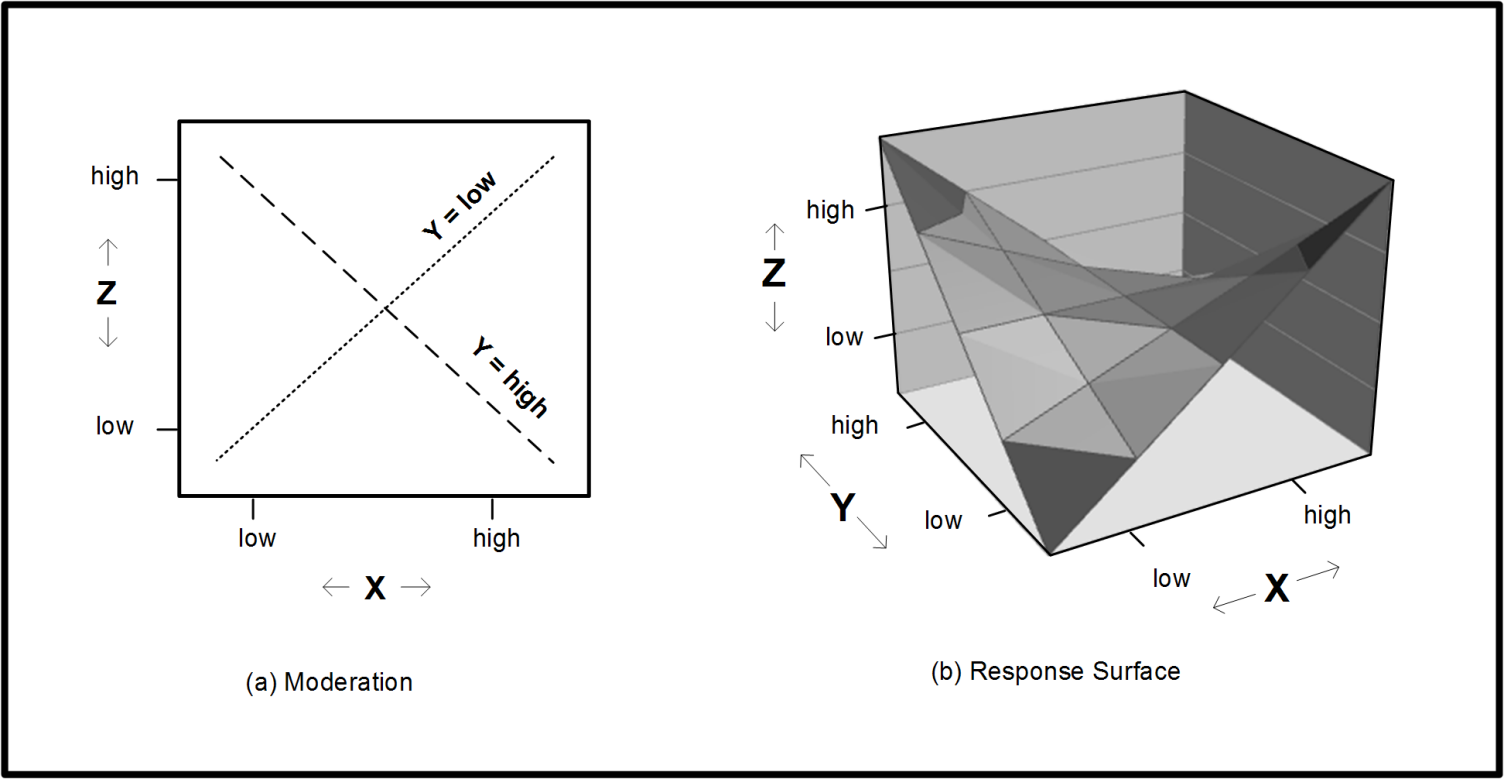
Illustration of identical results plotted on (a) a moderation graph, and (b) a response surface. In the moderation graph, X is the focal variable, Y is the moderator variable, and Z is the dependent variable. In the response surface graph, X is the first focal variable, Y is the second focal variable, and Z is the dependent variable.

To measure non-linear, disaggregated change, we rely on response surface analysis, which is a form of moderation analysis. In a typical moderation analysis, there is a focal variable (X), a moderator variable (Y), and an outcome (Z). Although X predicts Z, the X–Z relationship is variable, such that Y predicts the magnitude and direction of the X–Z relationship. In response surface analysis, a similar relationship between three variables is posited, but there are two differences. First, consider the moderator relationship shown in the upper part of Fig 1. On the left side, there is a familiar illustration of a three-variable relationship, where fixed values of Y, the moderator variable, are plotted. On the right side, the same relationship is illustrated in a response surface. Here, Y is no longer represented using discrete lines. Instead, it is represented on the dimension that recedes from the viewer. Thus X and Y are both plotted on axes, and are given equal status, while Z remains on the vertical axis, but it is now on a three-dimensional plane.

**Fig 2:**
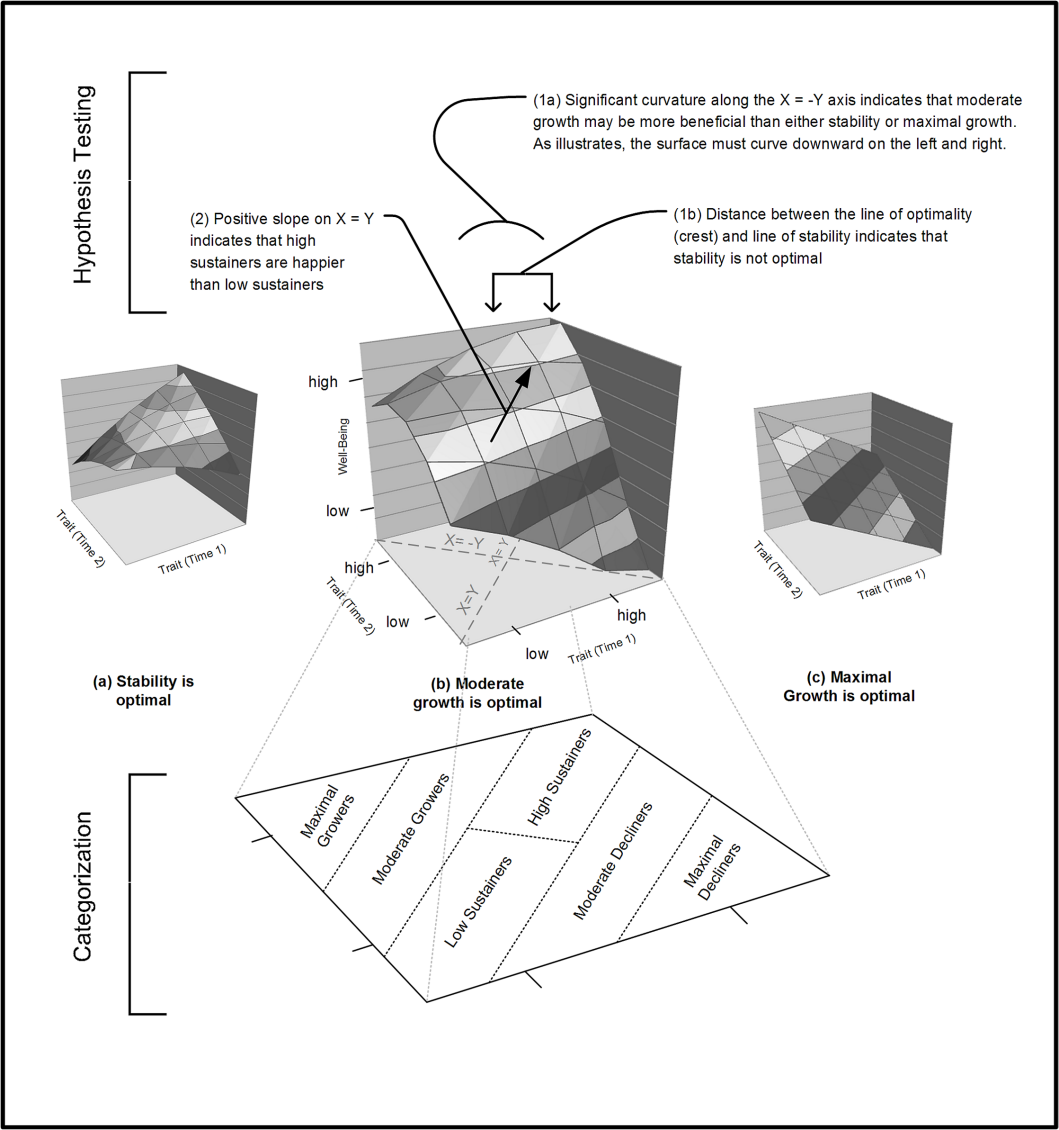
Hypothetical response surfaces supporting the hypotheses that (a) stability is optimal, (b) moderate growth is optimal, or (c) maximal growth is optimal.

Second, the theoretical focus is not on variation in outcomes based on linear variation in Y, which is the typical stance in a moderation analysis. Instead, the theoretical focus is on the congruence between X and Y. Congruence is achieved when X and Y are identical in value. Thus, instead of plotting the outcome when Y = +1 SD or Y = -1 SD, the researcher focuses on the line representing the outcome when Y = X. Researchers typically hypothesize that congruence causes an optimal outcome. For instance, a researcher may hypothesize that job satisfaction is at its peak when employee’s desired level of autonomy is congruent with the level of autonomy bestowed by the organization. If this hypothesis holds, there should be a ridge along the line X = Y, as illustrated in Fig 2 (see a), indicating that Z reaches its highest value when X and Y are congruent.

Hypotheses concerning stability can also be tested with response surfaces, because stability is a form of congruence. In Fig 2, consider X to represent extraversion at time 1 (in a two-wave study), Y to represent extraversion at time 2, and Z to represent well-being. Because X and Y both represent the same variable, X = Y is the line of stability (instead of the line of congruence). People with identical extraversion scores at time 1 and time 2 have their well-being scores plotted on this line. To its left are people whose extraversion increased, and to its right are people whose extraversion declined.

Because we are interested in change, these areas are more relevant to the current study than the line of congruence. Given a starting trait level and an absolute change value, one can use this graph to compute a predicted well-being score. For instance, if you are interested in a person whose starting trait score is one, and who changed by one point (i.e., final score = 1 + 1 = 2), locate one on the X axis and two on the Y axis. Now imagine lines extending perpendicularly from these axes, and note the point at which they intersect. The height of the surface at this point denotes the person’s predicted well-being score.

There are no a priori categories on the response surface. For clarity we create five categories to capture the primary configurations of continuity and change. As shown at the bottom of Fig 2, *high sustainers* have the same high level of extraversion at both time points, and *low sustainers* have the same low level of extraversion at both time points. *Moderate growers*, *maximal growers*, *moderate decliners*, and *maximal decliners* experience unique magnitudes and directions of change, as illustrated.

From left to right in Fig 2, we show three possible configurations of a response surface that represents surfaces that are pertinent to the current study. With well-being plotted on the Z axis, the surface (b) corresponds to the Goldilocks hypothesis. In this surface, Moderate Growers report the highest level of well-being.

The hypothesis testing procedure is illustrated at the top of Fig 2. To support hypothesis 1 (the hypothesis of moderate growth) we must reject surfaces (a) and (c), and we must find significant curvilinearity along the line X = -Y (i.e., the line that is perpendicular to the line of congruence). We must also show that that line of optimality distinctly differs from the line of stability, thus falsifying the hypothesis that stability is optimal. To do so, we must show that there is a distance greater than zero between the point (0, 0) and the equivalent point on the line of optimality. We must also show that the line of optimality runs roughly parallel to the line of stability; there is no discrete cutoff, but if the line of optimality is almost perpendicular, the surface would be altogether different from what we have hypothesized. These tests are conventional in response surface analyses. For details on the derivation of these tests, see [79,80]. For the details on the bootstrap procedure used to generate a confidence interval around the inter-line distance, see [81].

To support hypothesis 2 (the hypothesis of positive stability) we must verify that the line of stability slopes upwards toward high sustainers, as illustrated in Fig 2. We reverse score the negative trait, neuroticism, so that an upward slope retains the same meaning across all analyses.

## Methods

### Ethics Statement

This study did not qualify as human-subjects research under Emory University’s institutional guidelines because it involved no intervention or interaction with humans, and no access to identifiable private information.

### Sample

Data are from the MIDUS survey’s main random-digit dialing (RDD) sample. In the first wave of MIDUS (MIDUS 1; 1995–96) data were collected from 3,487 non-institutionalized English-speaking adults in the coterminous United States, ages 25–74. Random-digit dialing procedures were used with multistage sampling design, involving equal-probability sampling in the first stage and stratified sampling in the second stage. The sample was stratified by age and sex, with oversampling of men between the ages of 65 and 74. Initial collection of data occurred via telephone interviews requiring about 45 minutes (response rate = 70%). Participants were then requested to fill two self-administered questionnaires: 3,034 respondents filled these questionnaires (response rate = 87%). In the second wave of data collection (MIDUS 2; 2004–2006), survey administrators contacted as many of the original respondents as possible, and invited them to participate in MIDUS 2. Of the main RDD participants, 2,257 participated in the MIDUS 2 phone interview, yielding a base longitudinal retention rate of 65% and a mortality-adjusted rate of 71%. In addition, 1,805 respondents completed the MIDUS 2 self-administered questionnaires (completion rate = 80%).

We compared participants who filled out self-administered questionnaires in 1995 only to those who filled out self-administered questionnaires at both time-points. There were no significant differences in extraversion (sociality and agency) and neuroticism. The two-wave participants were marginally lower than the one-wave participants in conscientiousness (*d* = -0.15), emotional well-being (*d* = -0.15), negative affect (*d* = -0.10), and psychological well-being (*d* = -0.10). However, these effect sizes can be considered small [82]. In addition, the mean age of two-wave participants was 1.8 years lower than the mean age for one-wave participants. Demographic differences were substantial: 88% males among one-wave compared with 45.1% among two-wave participants, 14.9% non-white one-wave compared with 7.4% non-white two-wave participants, and 40.9% unmarried among one-wave compared with 32.2% two-wave participants (see also [83]). The demographic characteristics of the analyzed sample are listed in Table 1.

**Table 1:** Demographic Characteristics of Participants (N = 1,725).

	Number	Percent
Gender		
Male	778	45.1
Female	947	54.9
Age (1995)		
25–34	301	17.4
35–44	416	24.1
45–54	453	26.3
55–64	365	21.2
65–74	190	11.0
Education (1995)		
Some Grade School	138	8.0
High School	479	27.8
Some College	509	29.5
College Graduate	599	34.7
Marital Status (1995)		
Never Married	173	10.0
Married	1169	67.8
Separated	34	2.0
Divorced	258	15.0
Widowed	91	5.3
Marital Status (2005)		
Never Married	126	7.3
Married	1170	67.8
Separated	23	1.3
Divorced	258	15.0
Widowed	146	8.5
Household Income (1995)		
$24,999 or less	315	18.3
$25,000-$49,000	477	27.7
$50,000-$74,999	314	18.2
$75,000 or greater	619	35.9
Household Income (2005)		
$24,999 or less	366	21.2
$25,000-$49,000	392	22.7
$50,000-$74,999	313	18/1
$75,000 or greater	654	37.9

### Measures

#### Personality Traits

Measures of the Big Five personality traits were included in the self-report questionnaire. Two facets of extraversion—sociality and agency—were measured. For the other traits, faceted measures were not used. Each trait was measured with the MIDUS adjectival scale [84], which was developed from a combination of trait lists and inventories [85–87].

Participants were asked how much specific adjectives could be used to describe themselves on a scale from 1 (*not at all*) to 4 (*a lot*). The adjectives were outgoing, friendly, lively, active, talkative (*extraversion–sociality*); self-confident, forceful, assertive, outspoken, and dominant (*extraversion–agency*); moody, worrying, calm (reverse scored), and nervous (*neuroticism*); and organized, responsible, hardworking, and careless (reverse scored) (*conscientiousness*). An additional item used only in MIDUS 2 was excluded in our analyses. The items were arranged such that consecutive pairs of items did not measure the same trait. Reliabilities for the whole MIDUS were generally high: sociality (.78 in 1995; .77 in 2005), agency (.81 in 1995; .81 in 2005), neuroticism (.76 in 1995; .74 in 2005), and conscientiousness (.58 in 1995; .60 in 2005). Reliabilities of .70 and higher are acceptable for scales of this length [88]. Because the conscientiousness scale evinced inadequate reliability, we computed factor scores for conscientiousness for each wave in SPSS using the regression method, inputting the four corresponding items. Factor scoring was done at the request of a reviewer. Readers should note that factor scoring entails that the wave-1 scale for conscientiousness does not perfectly align with the wave-2 conscientiousness scale. A person with the same score on both wave-1 and wave-2 conscientiousness may not have been perfectly stable, but the discrepancy is likely small enough to be meaningless.

The factorial invariance of the MIDUS Big Five trait scales (excluding agency) across waves 1 and 2 was examined by Zimprich, Allemand, & Lachman [89]. Strict measurement invariance was found in terms of factor loadings and factor count. Following Little [90], we tested *agency* for invariance, using a single-factor model with the fixed-factor method. In this method, the variance of the wave-1 latent factor is set to 1.0 in the configural and weak factorial models; and the variance of both wave-1 and wave-2 latent factors is set to 1.0 in the strong factorial model. The CFI for these three models was .981, .980, and .971 respectively. Based on a difference threshold of .01 [91], these CFI values suggest strong factorial variance.

#### Subjective Well-Being

Emotional well-being and psychological well-being were examined as outcomes. The terms *hedonic well-being* and *eudaimonic well-being* are sometimes uses as synonyms for these two dimensions respectively. These two dimensions of well-being have been demonstrated to be substantively distinct [92] and factorially distinct [92,93], having arisen from two distinct philosophical traditions [68,94].

For emotional well-being, two measures were derived. The primary measure, which we simply term *positive affect*, was computed by equally weighting the measures of positive affect and life satisfaction, and then summing them. Positive affect was measured by asking participants to indicate on a scale from 1 (*none of the time*) to 5 (*all of the time*) how frequently during the past thirty days they felt six kinds of positive affect. These six kinds were “cheerful,” “in good spirits,” “extremely happy,” “calm and peaceful,” “satisfied,” and “full of life.” Life satisfaction was measured using a single measure of global satisfaction with one’s life [95]. It asked participants to “rate their life overall these days” on a scale from 0 (*worst possible life overall*) to 10 (*best possible life overall*). Because positive and negative affect are orthogonal under some moderating conditions, and highly correlated under other conditions, we did not compute an affect balance score. Negative affect was measured in the same manner as positive affect. The six kinds of negative affect were “so sad nothing could cheer you up,” “nervous,” “restless or fidgety,” “hopeless,” “that everything was an effort,” and “worthless”. Because of high negative skewness, we applied a square-root transformation to the negative-affect scores. The internal consistencies of the positive and negative affect scales were .91 and .83 respectively in 1995, and .91 and .82 respectively in 2005.

Six scales of psychological well-being (PWB) were used: Self-Acceptance, Environmental Mastery, Positive Relations with Others, Personal Growth, Purpose in Life, and Autonomy [68]. Each scale consisted of three items, some of which were reverse scored. Respondents indicated on a scale of 1 (*strongly agree*) to 6 (*strongly disagree*) whether they agreed that an item described how they thought and felt. Examples of items are: “I am not afraid to voice my opinions, even when they are in opposition to the opinions of most people” (*autonomy*), “In general, I feel I am in charge of the situation in which I live” (*environmental mastery*), “I have the sense that I have developed a lot as a person over time” (*personal growth*), “Most people see me as loving and affectionate” (*positive relations*), and “I enjoy making plans for the future and working to make them a reality” (*purpose in life*). Scores were summed and coded such that a higher score represented higher well-being. The internal consistency of the overall PWB scale was .76 in 1995 and .73 in 2005.

We tested each of these three factors for factorial invariance using the method described earlier. In the PWB models, we used the summed scores of each dimension, provided in the MIDUS dataset, as indicators of a first-order model. For PA (including the life satisfaction item), the CFI for configural, weak factorial, and strong factorial models was .917, .916, and .914 respectively. For NA, the CFI for configural, weak factorial, and strong factorial models was .902, .904, and .900 respectively. For PWB, the CFI for configural, weak factorial, and strong factorial models was .963, .961, and .909 respectively respectively. Given the failure of the strong factorial invariance test for PWB, we allowed three pairs of intercepts (positive relations, self-acceptance, and purpose in life) to be unequal across waves. The resultant CFI was .956, indicating partial strong factorial invariance [90].

#### Covariates

Transitions through social roles across the life-course and organismic maturation appear to cause changes in both well-being and personality [96–98]. To control for these confounding factors, we entered age (standardized) as a covariate in our analyses. (Age itself cannot cause an outcome, and it is therefore a proxy.) Because prior evidence indicates curvilinear associations between age and pertinent outcomes, we also entered age^2^ and age^3^. In addition, we investigated if age^4^ was a statistically significant predictor of any outcome. It was statistically significant in regressions modeling PWB, and was included in those regressions. Well-being at Time 1 was mean-centered and entered as a covariate in order to model change in well-being across the two waves of MIDUS. Other demographic covariates were not entered because such factors are known to be causes of distinct *levels* of traits and well-being, but they may not produce distinct *trajectories* of traits and well-being.

### Data Analysis

We conducted hierarchical regressions using the HCREG–HC 3 procedure, which corrects for heteroscedasticity [99]. Well-being was entered as the dependent variable, and separate regressions were run for each type of well-being. In step 1, we entered the time 1 value of well-being and all the covariates and the linear terms for the independent variable, namely, trait level at MIDUS 1 (x) and trait level at MIDUS 2 (y). In step 2, we entered the quadratic terms, x^2^, xy, and y^2^. In step 3, we entered the cubic terms x^3^, x^2^y, xy^2^, and y^3^. The HCREG procedure conducts an F test to determine if the addition of coefficients at every step explains a significant increment of variance, which is analogous to testing the incremental R^2^ in a hierarchical regression. We thus determined if the best model was linear, quadratic, or cubic.

Using combinations of coefficients in quadratic models, we then conducted significance tests for slope and curvature along the line of stability, which is X = Y, and along the line perpendicular to this line, which is X = -Y. The test of curvature along X = -Y was used to determine if there was a line of optimality (i.e., a crest), which would support hypothesis 1 (the hypothesis of moderate growth). The test of slope significance along X = Y was used to test hypothesis 2 (the hypothesis of positive stability).

A confidence interval is estimated for the term-*p*_*10*_ */(p*_*11*_ *+1)*, which denotes the displacement between the line of optimality and the line of stability at (0,0), where *p*_*10*_ equals the intercept and *p*_*11*_ equals the slope of the line of optimality along the XY plane (i.e., not along the Z axis). If the 95% bias-corrected confidence interval for the term *p*_*10*_ */(p*_*11*_ *+1)* excludes zero, the null hypothesis is rejected, which the first sub-test for hypothesis 1. This is akin to a one-tailed test with an alpha level of .025.

A confidence interval is also estimated for *p*_*11*_. There are three possible outcomes. First, if the confidence interval narrowly straddles one (e.g., 0.95–1.05), the line of optimality is roughly parallel to the line of stability, indicating that moderate growth is uniformly beneficial. Second, if the interval includes one but does not narrowly straddle it, we can neither reject the experimental hypothesis that the two lines are parallel nor can we reject the null hypothesis that they are intersecting. Third, if the interval excludes one, we can conclude that the two lines intersect. If this intersection occurs above the midpoint, we can conclude that moderate growth is optimal for people whose initial trait score is below the midpoint or at the midpoint of the scale, but that at the upper end of the scale, there is uncertainty about such an effect. The uncertainty arises because stability appears to be beneficial at the upper end of the scale but a ceiling effect cannot be ruled out.

#### Robustness checks

Two robustness checks were conducted. First, we filtered the dataset to only include participants who had the potential to attain large increases. Using quadratic regression with graphs, we examined whether there was a curvilinear effect of trait change on well-being. No covariates were entered in these analyses because the goal was to visually analyze the graphs.

Second, we removed outliers from the dataset, and conducted piecewise regression analyses to examine whether the slope for participants who underwent substantial positive change differed from the slope for participants who did not undergo substantial positive change. Substantial positive change was defined as having a change score that was more than one standard deviation above the mean. The covariates that were used in the main analysis were also used in these analyses.

## Results

**Fig 3:**
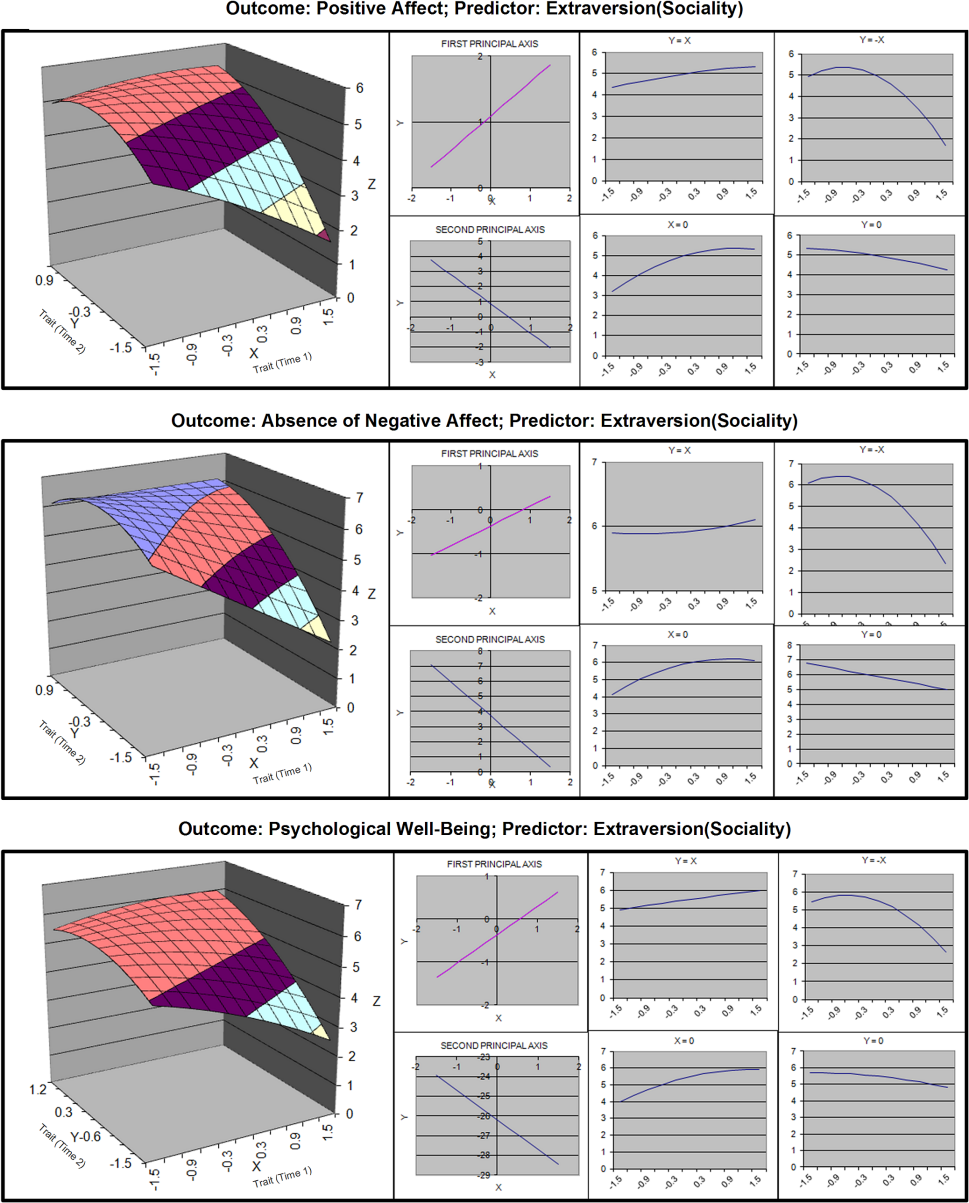
Response surfaces for polynomial analyses of sociality and well-being.

**Fig 4:**
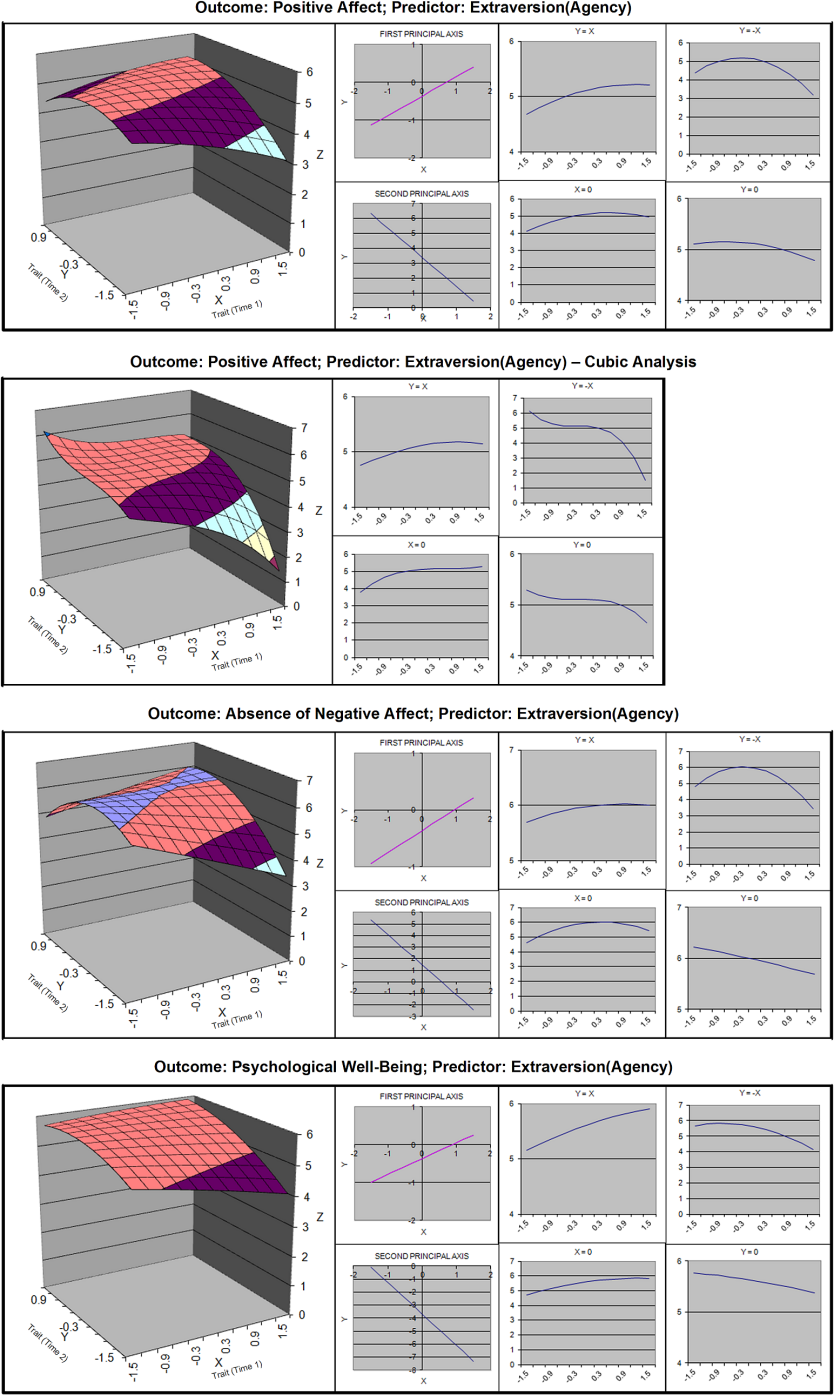
Response surfaces for polynomial analyses of agency and well-being.

**Fig 5:**
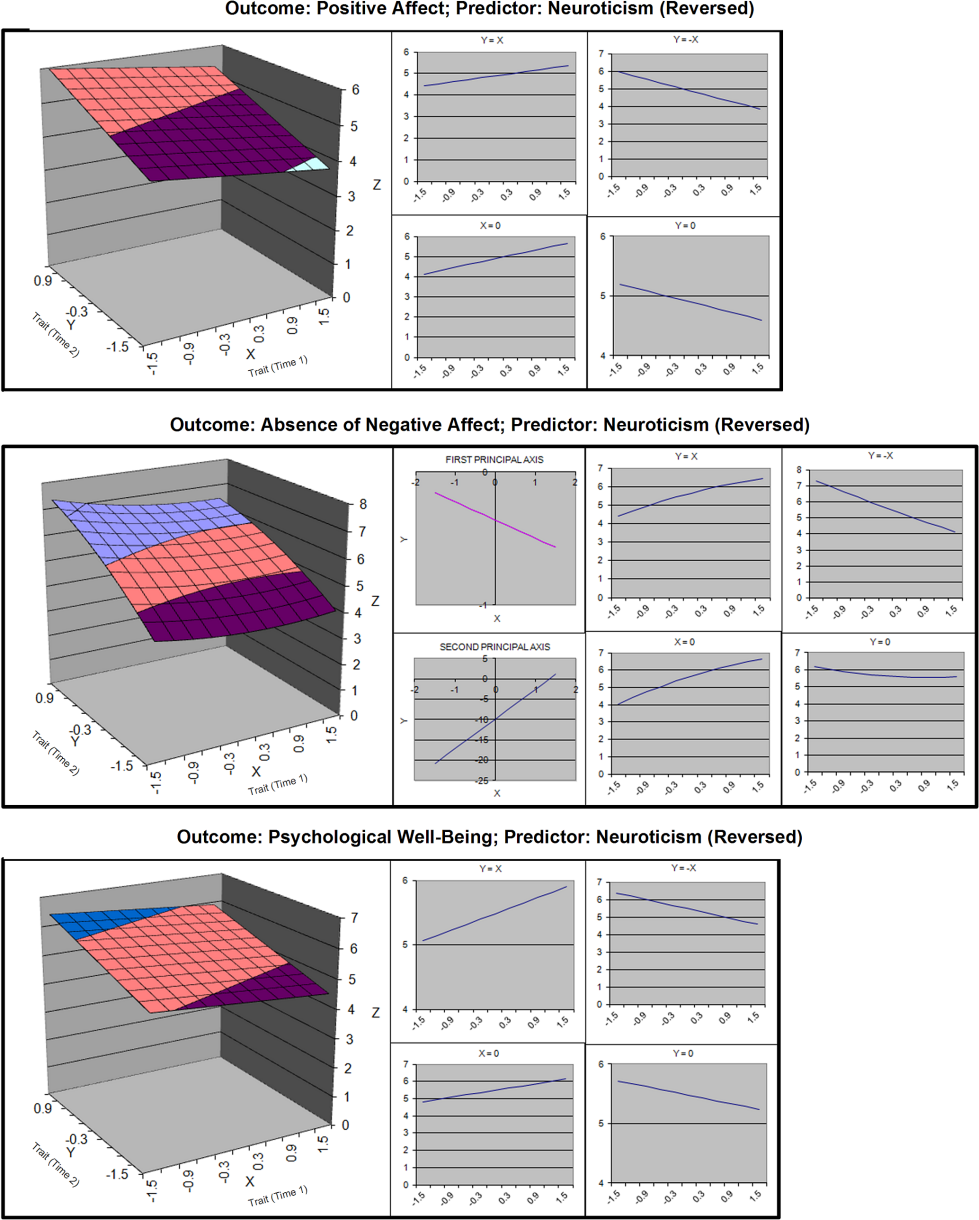
Response surfaces for polynomial analyses of neuroticism and well-being.

**Fig 6:**
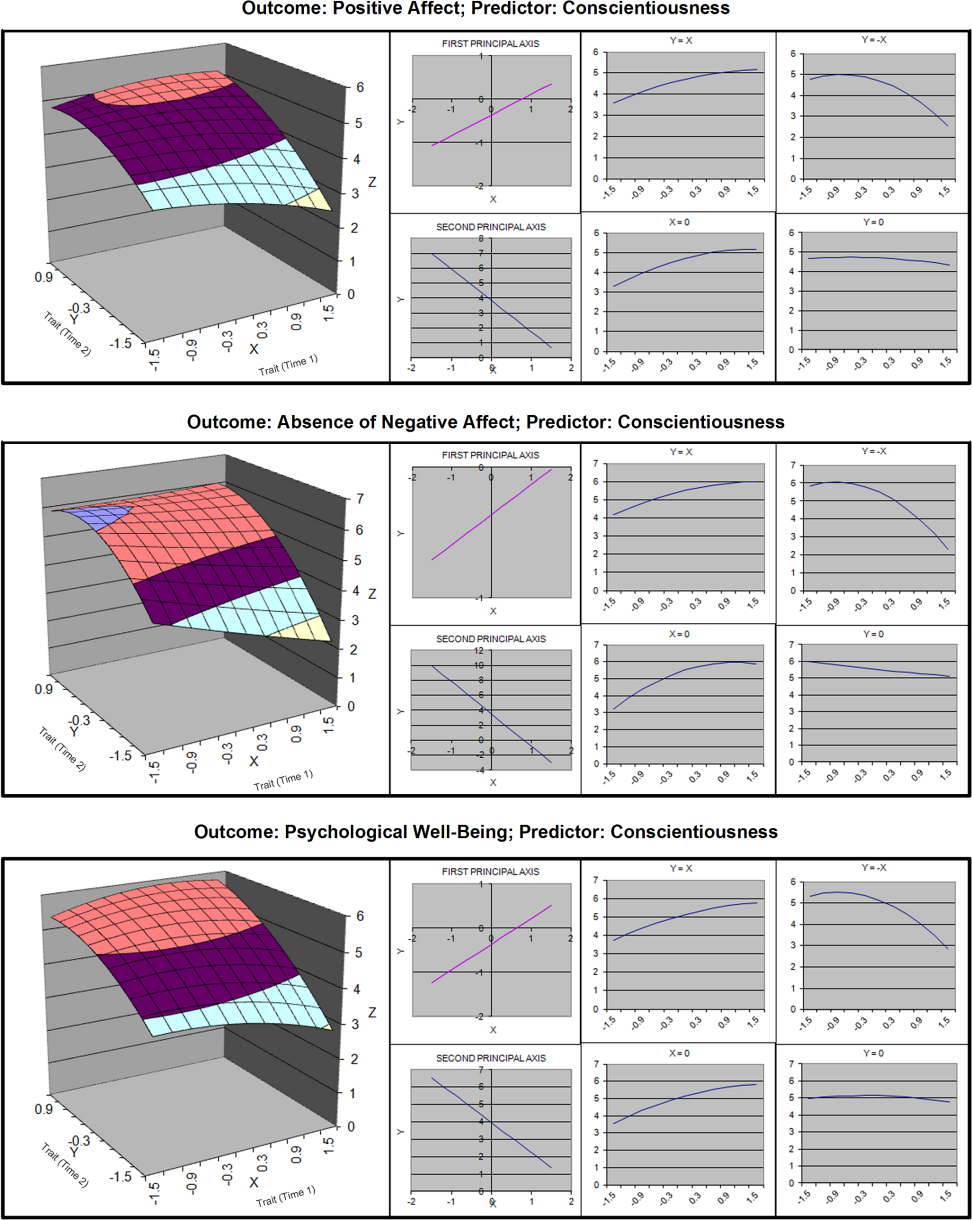
Response surfaces for polynomial analyses of conscientiousness and well-being.

**Fig 7:**
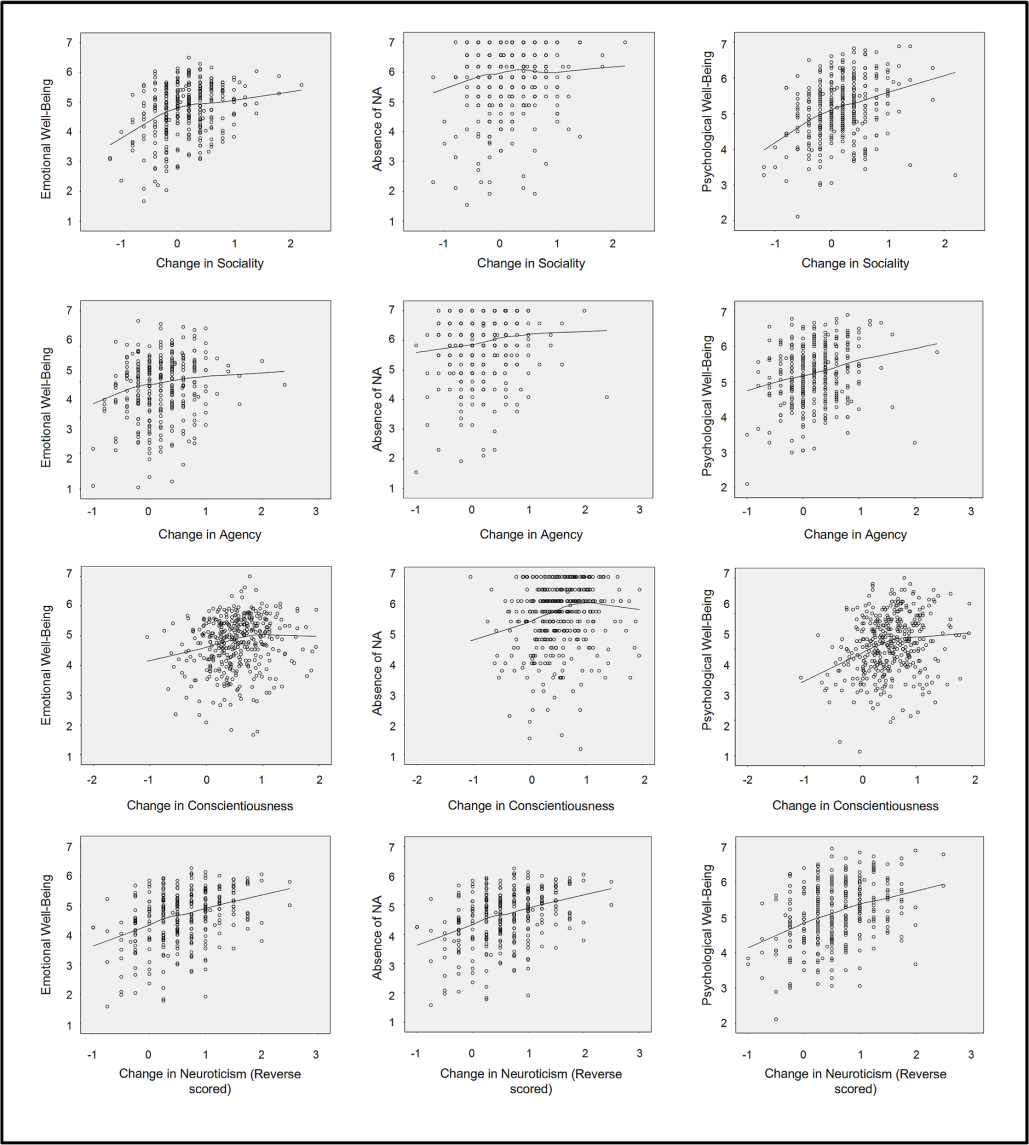
Scatterplots of Trait Change (X axis) and Well-Being (Y axis) among participants in the lowest trait quartile at Time 1. The lines of best fit are from lowess regressions using the Epanechnikov kernel with 75% of points fitted.

Descriptive statistics and correlations between predictor variables and outcome variables can be found in Tables 2 and 3 respectively. Response surface analyses, which are derived from polynomial regressions, are displayed in Figs 3–7. The figures were generated using a template by Edwards [100]. In the case of positive affect predicted by agency, the cubic polynomial terms were statistically significant. Thus, in Fig 4, a cubic plot supplements the quadratic plot. However, the significance of the cubic terms is likely the result of outliers. The lowess regressions, which we discuss later, show that the PA–agency association resembles the other associations.

**Table 2:** Trait and Well-Being Levels of Participants (N = 1,725).

	MIDUS 1		MIDUS 2	
	Mean	SD	Mean	SD
Traits (-1.5 to 1.5 scale)				
Extraversion				
Sociality	.70	.56	.60	.59
Agency	.22	.66	.14	.67
Neuroticism	-.26	.67	-.41	.64
Conscientiousness	.94	.44	.95	.46
Well-Being (1 to 7 scale)				
Emotional well-being	5.03	0.84	5.05	.85
NA (Reversed)	5.87	1.11	5.88	1.07
Psychological well-being	5.55	0.77	5.52	.82

*Note*. *NA* = negative affect. NA scores were square-root transformed and reversed to produce scores for NA. Emotional well-being is the sum of positive affect and life satisfaction. Conscientiousness was computed using factor scores.

**Table 3:** Inter-Correlations of Predictor and Outcome Variables.

	Age	E1	E2	A1	A2	N1	N2	C1	C2	PA1	PA2	NA1	NA2	PWB1	PWB2
Age	1	.005	.034	-.016	.021	-.195**	-.202**	.031	-.020	.188**	.172**	.161**	.127**	.048*	.046
E1		1	.683**	.505**	.405**	-.153**	-.128**	.260**	.186**	.324**	.234**	.201**	.102**	.392**	.322**
E2			1	.410**	.526**	-.123**	-.204**	.205**	.266**	.287**	.399**	.157**	.239**	.311**	.476**
A1				1	.703**	-.071**	-.067**	.218**	.175**	.148**	.144**	.105**	.076**	.286**	.276**
A2					1	-.066**	-.084**	.154**	.234**	.126**	.218**	.088**	.140**	.230**	.369**
N1						1	.627**	-.186**	-.117**	-.465**	-.314**	-.596**	-.392**	-.471**	-.330**
N2							1	-.156**	-.184**	-.332**	-.433**	-.438**	-.559**	-.355**	-.447**
C1								1	.611**	.283**	.228**	.173**	.147**	.400**	.321**
C2									1	.242**	.302**	.168**	.222**	.294**	.402**
PA1										1	.546**	.607**	.340**	.580**	.405**
PA2											1	.366**	.597**	.405**	.599**
NA1												1	.493**	.546**	.361**
NA2													1	.355**	.504**
PWB1														1	.582**
PWB2															1

Note

* p < .05

** p < .01

*** p < .001.

The complete polynomial regression results are in a supporting information file, and include only coefficients that were kept in the analysis (**S1 File)**. We caution that the individual regression coefficients (except for covariates) have little meaning in isolation.

Hypothesis 1 was that moderate growth would cause the highest level of individual well-being. We tested this hypothesis by first determining if there was curvature along the line of stability, and then testing if the line of optimality was significantly displaced from the line of stability, measured along X = -Y. Estimates of the slopes and curvature coefficients of the lines of stability and its orthogonal are displayed in Table 4, and linear plots of these lines can be found in Figs 3–6. Estimates of displacement between the line of stability and the line of optimality are displayed in Table 5.

**Table 4:** Trait Change Analyses: Estimates of the Slopes and Curvature Coefficients of Line of Stability and Its Orthogonal.

	EWB 2		NA 2 (reversed)		PWB 2	
	B	CI_95%_	B	CI_95%_	B	CI_95%_
Sociality						
X = Y Slope	0.33	0.19, 0.46	0.07	-0.11,0.27	0.35	0.24,0.46
X = Y Curvature	-0.06	-0.17,0.04	0.04	-0.11,0.17	-0.02	-0.11,0.06
X = -Y Slope	-1.08	-1.30,-0.85	-1.25	-1.58,-0.94	-0.94	-1.15,-0.72
X = -Y Curvature	-0.73	-1.13, -0.39	-0.77	-1.40,-0.14	-0.65	-1.05,-0.22
Agency						
X = Y Slope	0.18	0.11,0.24	0.11	0.03,0.18	0.25	0.20, 0.31
X = Y Curvature	-0.08	-0.16, -0.00	-0.06	-0.16,0.04	-0.04	-0.10, 0.03
X = -Y Slope	-0.39	-0.54, -0.25	-0.46	-0.66,-0.27	-0.52	-0.66, -0.37
X = -Y Curvature	-0.60	-0.97,-0.23	-0.82	-1.30,-0.28	-0.32	-0.69, 0.06
Conscientiousness						
X = Y Slope	0.46	0.15, 0.72	0.48	0.01,0.87	0.75	0.53,0.96
X = Y Curvature	-0.13	-0.28,0.05	-0.15	-0.37,0.11	-0.26	-0.38,-0.13
X = -Y Slope	-0.63	-1.04,-0.24	-1.16	-1.76,-0.60	-0.60	-0.95,-0.25
X = -Y Curvature	-0.46	-0.97,-0.03	-0.79	-1.54,-0.05	-0.55	-1.03,-0.09
Neuroticism						
X = Y Slope	0.31	0.25,0.38	0.68	0.57,-0.81	0.29	0.23,0.35
X = Y Curvature			0.10	-0.19,0.00		
X = -Y Slope	-0.72	-0.84,-0.59	-1.06	-1.28,-0.86	-0.60	-0.72,-0.48
X = -Y Curvature			0.04	-0.30,0.34		

*Note*. The bias-corrected confidence intervals were computed using bootstrapping. Blank cells indicate regressions in which quadratic terms were excluded because they explained no additional variance. NA was square-root transformed prior to analysis in order to minimize skewness.

* p < .05

** p < .01

*** p < .001.

**Table 5:** Estimates and confidence intervals of displacement and parallelism between the lines of stability and optimality.

	Displacement		Parallelism	
	*-p10/(p11+1)*	CI_95%_	*p11*	CI_95%_
Sociality				
EWB	-0.73	[-1.24, -0.47]	0.52	[0.28,0.79]
NA (R)	-0.71	[-1.68,-0.40]	0.45	[0.12,0.73]
PWB	-0.75	[-2.08,-0.41]	0.66	[0.27,1.07]
Agency				
EWB	-0.34	[-0.76, -0.18]	0.51	[0.20, 0.81]
NA (R)	-0.25	[-0.53,-0.13]	0.38	[0.14,0.58]
PWB	-0.82	[-5.46,-0.35]	0.42	[-0.20,0.93]
Conscientiousness				
EWB	-0.77	[-5.80,-0.12]	0.53	[-0.47,2.55]
NA (R)	-0.68	[-1.70,-0.33]	0.31	[-0.08,0.70]
PWB^1^	-0.53	[-8.84,0.28]	1.02	[-1.91,9.74]
Neuroticism (Rev.)				
EWB	—		—	
NA (R)	-3.29	[-11.83,-1.61]	-0.13	[-0.49,0.20]
PWB	—		—	

*Note*. The bias-corrected confidence intervals were computed using bootstrapping. EWB = emotional well-being. NA (R) = negative affect (reversed). PWB = psychological well-being. NA was square-root transformed prior to analysis in order to minimize skewness.

### Main Analysis

#### Sociality and Agency

For both facets of extraversion, curvature was found along the X = —Y line for all well-being outcomes (see Table 4). The displacement was significant (see Table 5), indicating there was a “just right” moderate amount of growth. The confidence intervals around the slopes did not include 1. Thus, the line of optimality generally converged with the line of stability at the upper end of the scale, which indicates that the “just right” amount pertains to people whose initial value was below or at the midpoint. For people at the upper end of the scale, stability may have been beneficial or a ceiling effect may have occurred.

#### Conscientiousness

For conscientiousness, curvature was found along the X = -Y axis for all three types of well-being. The displacement was significant for PA and absence of NA. For PWB, zero fell within the bounds of the confidence interval, but a post hoc analysis revealed that the displacement test would have passed had the CI upper bound been set at 94.65% (*z* = 1.612), instead of 97.5%. Given this negligible difference, we interpret the displacement as a Goldilocks effect. In the conscientiousness–NA surface, the line of optimality intersected the line of stability at the upper end of the trait scale. Thus, for people at the upper end of the scale, stability may have been beneficial or a ceiling effect may have occurred. In the conscientiousness–PA and conscientiousness–PWB surfaces, the parallelism coefficient had a confidence interval straddling one. Therefore, it is plausible that the two lines were parallel, and that moderate growth was optimal along the entire spectrum of initial values. However, the interval was wide, indicating uncertainty.

#### Neuroticism

Analyses of neuroticism did not reveal the expected pattern of results. For both EWB and PWB, the linear model was best fitting. Response surfaces revealed that monotonic negative change in neuroticism was associated with greater EWB and PWB. For the NA surface, the curvature coefficient was positive, which suggested that large amounts of change were exponentially more beneficial than small amounts of change. However, the curvature coefficient was non-significant. Overall, the surface indicated that it was optimal to have a moderate-to-low level of neuroticism at time 2 (Fig 5). Whether this level was attained through change or absence of change did not seem to matter.

The mean stability–optimality displacement across all Goldilocks cases was 0.62, and the average standard deviation for each trait was approximately 0.60. Thus, in cases where a “just right” amount of positive change was found, the right amount for a person whose initial value was at the midpoint was approximately one standard deviation, albeit measured in a diagonal—not lateral—fashion on the response surface.

### Robustness Checks

Because our primary concern was positive trait change, which entails *increases* in trait strength, we conducted a robustness check that was limited to participants whose initial trait score was in the lowest quintile of the sample, and thus had the potential to increase by a small or large amount. Participants in the higher quintiles were closer to the scale ceiling, and thus had less potential to display positive change on the MIDUS scales.

In these analyses, we entered the same outcomes, but did not enter the full set of polynomial terms. Rather, we used change scores as our independent variable. We then plotted the line of best fit using lowess regressions with the Epanechnikov kernel based on 75% of points. The results are shown in Fig 7. The X-axis on the conscientiousness plots is distinct because factor scores were used for this trait. These results comport with results of the response-surface analysis in one sense: moderate change and extreme change were separated at an inflection point. They contrast with the response surface analysis, however, inasmuch as the line after the inflection point does not slope downward except in the pairing of conscientiousness and absence of NA. In the remaining cases, the line flattened or sloped upward at a shallower angle. The lowess regression results do not show an inverted-U. Since these analyses rely on change scores, however, they are less reliable than the response surface analyses. The low reliability is a weakness that makes this analysis less informative.

To tackle the problem of outliers, we conducted a second robustness check. We began by eliminating outliers from the dataset. We standardized the scores for trait, trait-change, and well-being variables, and following Tabachnick and Fidell [101], removed cases with z-scores less than -3.29 or greater than 3.29 on any of these variables, because 99.9% of standardized scores fall between -3.29 and 3.29.

**Fig 8:**
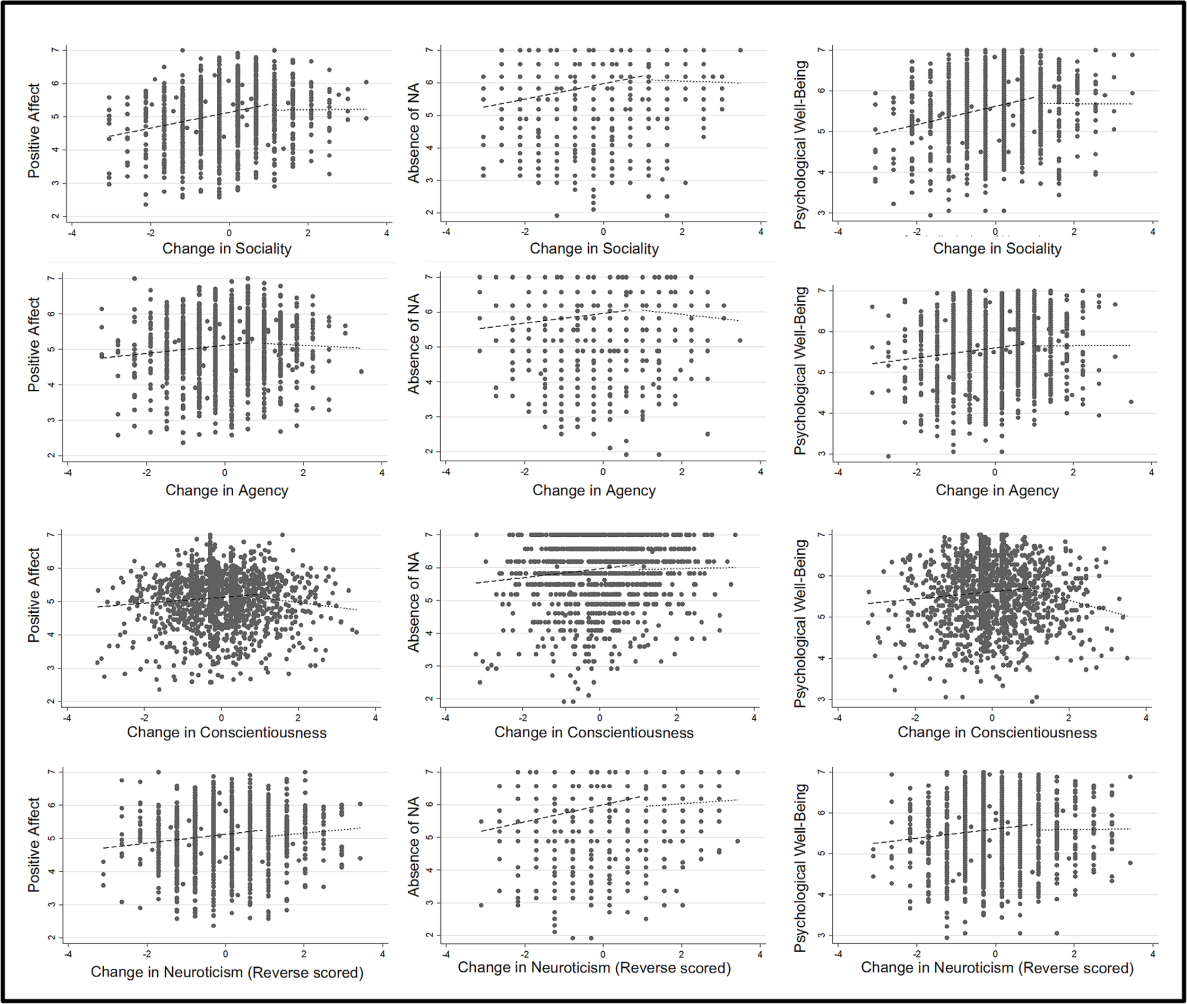
Scatterplots of Standardized Trait Change (X axis) and Well-Being (Y axis) with outliers removed. Lines represent the results of piecewise regression analyses.

We then conducted a piecewise regression to examine whether the change–wellbeing association differed between two discrete categories: people who experienced one standard deviation or less of positive trait change, and people who experienced more than one standard deviation of trait change. We refer to these categories as *moderate* and *large* changers respectively. (The first category included all participants with a standardized change of score of 1 or less—participants with negative trait change or stability were included, and thus the term *moderate* is only used for convenience.) In the piecewise regression, which was conducted in Stata, we regressed well-being at time 2 on the trait change score, controlling for the same covariates as in the main analysis, and computed the regression slope separately for the moderate and large categories. In Table 6, these slopes are displayed in the rows labeled *moderate* and *large* respectively. The associated scatterplots and lines of best fit are displayed in Fig 8, where the contrast between the slopes for moderate and large changes is evident. (The scatterplots are bivariate plots without adjustment for covariates.) The full piecewise regression results can be found in a supporting file (**S2 File**). The row labeled *ΔSlope* displays the difference between these two slopes, and the row labeled *Interval* displays the distance between the predicted well-being score of a person with a standardized change score of 1 and person with a standardized change score marginally below 1. Negative values here indicate that the person with the marginally lower trait-change score had a higher predicted well-being score.

**Table 6:** Trait Change Analyses: Piecewise Regression with Moderate Changers and Large Changers.

	EWB Wave 2		NA Wave 2 (reversed)		PWB Wave 2	
	B	SE_B_	B	SE_B_	B	SE _B_
Sociality						
Moderate	0.23***	0.02	0.23***	0.03	0.22***	0.02
Large	0.06	0.08	-0.01	0.11	0.01	0.09
ΔSlope	-0.17*	0.08	-0.24*	0.12	-0.20*	0.09
Interval	-0.12^†^	0.07	-0.07	0.10	-0.11	0.07
Agency						
Moderate	0.10***	0.02	0.13**	0.03	0.12***	0.03
Large	0.01	0.08	-0.08	0.11	0.04	0.08
ΔSlope	-0.12	0.08	-0.20^†^	0.12	-0.08	0.09
Interval	-0.03	0.06	-0.03	0.09	-0.06	0.07
Conscientiousness						
Moderate	0.08***	0.02	0.13***	0.03	0.08**	0.03
Large	-0.05	0.08	0.10	0.11	-0.18*	0.09
ΔSlope	-0.13	0.08	-0.03	0.12	-0.27**	0.09
Interval	0.02	0.08	-0.06	0.11	-0.05	0.09
Neuroticism (Rev.)						
Moderate	0.18***	0.02	0.30***	0.03	0.15***	0.03
Large	0.21**	0.07	0.18^†^	0.10	0.10	0.07
ΔSlope	0.03	0.07	-0.12	0.10	-0.06	0.07
Interval	-0.12^†^	0.07	-0.24*	0.09	-0.08	0.07

*Note*. Absence of NA was square-root transformed prior to analysis in order to minimize skewness.

^†^ p < .10

* p < .05

** p < .01

*** p < .001.

For three traits—sociality, agency, and conscientiousness—the piecewise results support the argument that positive change confers benefits up to an inflection point. For moderate changers—those below the Goldilocks point—the slopes were positive and statistically significant in all nine analyses, indicating that incremental increases in positive trait strength (up to this point) predicted incremental increases in well-being. But for large changers, the slopes in eight out of nine cases did not differ from zero. In five of the nine analyses, the slopes in the moderate range and large range were significantly different from each other, but the non-significance in the other four cases is less pertinent than the fact that the slopes in the large range did not significantly differ from zero. Although the slopes in the large range were negative in some cases, they did not reach statistical significance (except in one case), which detracts from the argument that excessive change may reduce well-being. However, the effect size may have been weak, and insufficient statistical power may have been an issue. The interval (between a person with a 0.999 SD change score and a 1 SD change score) was negative in eight out of nine cases, which supports the argument that excessive change may reduce well-being, but in a majority of cases the interval was not significantly different from zero.

For neuroticism, the results were somewhat similar. In the case of PWB, the slope was positive only in the moderate range. However, in the other two cases (PA and NA), the slope was also significantly positive in the large range. Additionally, the interval between the regression lines was negative in all three cases (albeit non-significant in one case), which suggests that attaining slightly more than one standard deviation of positive trait change was less beneficial than attaining slightly less than one standard deviation of positive trait change. However, declines in neuroticism of any magnitude were predictive of greater PA, which comports with the response surface analyses. Since these analyses are based on difference scores, it is understandable that they partially diverge from the results of the response surface analysis. The results of the response surface analysis likely have greater reliability, and should be preferred.

### Analysis of Trait Stability

Hypothesis 2 was that the line of stability would have a significantly positive slope, indicating that high sustainers had higher well-being than low sustainers. In the case of sociality and agency, we found a positive slope in all analyses except Sociality–Absence-of-NA, where the slope was positive but non-significant (see Table 4). For conscientiousness, the slopes were similarly positive for all three outcomes. However, the curvature coefficient was significantly negative for conscientiousness–PWB (Fig 6), giving the response surface a dome shape. Thus, among sustainers, there was also a “just right” level of conscientiousness, above which there was no discernible effect. In the case of neuroticism (reverse scored), the slope was also positive in all three outcomes. Although the curvature coefficients were largely non-significant, they were almost uniformly negative, which suggests that the benefits of being a moderate sustainer (rather than a low sustainer) are large, but the benefits of being a high sustainer (rather than a moderate sustainer) are small.

## Discussion

The goal of this study was to reconcile two theoretical perspectives on positive trait change: one extolling its benefits, the other its costs. We hypothesized that moderate positive trait change was the “just right” amount that would predict the highest level of well-being. When the benefits of positive trait from one process (i.e., self enhancement) and drawbacks from another process (i.e., violation of self verification) accrue at different rates, there is an inflection point where the overall outcome ceases to be beneficial. Response surface analyses revealed that moderate increases in sociality, agency, and conscientiousness were optimal, as hypothesized. Thus, the evidence supports the Goldilocks hypothesis for these three traits.

The detection of Goldilocks effects (and other curvilinear effects) hinges on the use of polynomial coefficients rather than linear coefficients. Prior studies of trait change solely used linear coefficients, and Goldilocks effects and other curvilinear effects were thus undetectable. As others have noted [70], the Goldilocks effect is a common feature in the positive psychology literature, and inattention to such effects may be limiting to progress when well-being is the outcome of interest. (We caution that polynomial coefficient analysis is necessary but insufficient for the detection of optimal levels. Post hoc analyses, such as those conducted here, must be used to detect optimal levels after significant polynomial coefficients have been found.)

It is unclear whether people experience a significant *loss* of well-being once they cross the optimal threshold, because the response surface analyses were only partially consistent with the piecewise regression analyses. The response surface analyses suggest that there are costs to excessive positive change, whereas the piecewise regression analyses suggest that there are neither costs nor benefits to excessive positive change. Nevertheless, both types of analyses indicate an inflection point. We consider the response surface analyses to be more reliable because they do not depend on differences scores, which are methodologically problematic. Future analyses with multiple waves of measurement will be useful in clarifying this ambiguity.

Change in neuroticism had a markedly different impact on well-being. Across all three well-being variables—including the case where some curvilinearity was detected—maximal decline was optimal, according to the response surface analyses. Put differently, there seems to be no point of diminishing returns to well-being when it comes to declines in neuroticism. The piecewise regression results contrasted with the response surface analyses in this case. Whereas the response surface analyses showed that declines in neuroticism were healthy across all three outcomes, the piecewise regressions showed one exception–when PWB was the outcome, large changes were not particularly beneficial. We once again interpret the response surface analyses as being more reliable. Nevertheless, these contrasting results leave some ambiguity about how substantial changes in neuroticism affect well-being. Future research on trait change should examine this connection in greater detail.

The contrast between neuroticism and the other traits indicates that researchers should be wary of measuring aggregate trait change instead of attending to each trait individually. The aggregation method, which was used by Human et al. [2], led to the conclusion that trait change uniformly caused negative outcomes. They also reported results for each trait, but their conclusions were largely drawn from their analysis of aggregated change (p. 6). This analysis glossed over the heterogeneity of effects demonstrated here.

We also hypothesized that positive stability would be beneficial, predicting that high sustainers would evince greater well-being than low sustainers, and this hypothesis was supported across all traits. For conscientiousness, we found one anomaly: moderate sustainers reported greater psychological well-being than low sustainers, but high sustainers did not report greater psychological well-being than moderate sustainers. The response surface peaked approximately halfway between moderate and high on the line of stability. However, this finding was not replicated for emotional well-being and NA. When it comes to PWB, it may be that there is an adequate level of conscientiousness that results in good psychological functioning.

McAdams and Olson [102] noted that sustainers are "often those who already show the dispositional signature associated with maturity—low neuroticism and high agreeableness, conscientiousness, and extraversion" (p. 7). Thus, sustainers may not only have benefited from stability but also from early attainment of a mature personality profile.

### Causal Claims and Limitations

Causal claims are warranted when threats to validity have been eliminated [103]. In the current study, our goal was to demonstrate that stability and change exert an effect on well-being, and we sought to eliminate all detectable threats to validity. We thus controlled for confounding factors and baseline levels of well-being. Because endogeneity can be a threat to validity, we also used two-stage least squares regressions with instrumental variables to verify that our results were robust. Specifically, we found instrumental variables for sociality and agency, but the two-stage least squares regression did not produce meaningfully different point estimates.

One weakness is that the current study relies on only two waves of measurement. The causal inferences are therefore less robust than those from panel studies with three or more waves. A third wave of MIDUS data is currently being collected and we plan to conduct confirmatory analyses when such data become available. Because the personality and well-being measures were not perfectly reliable, there may have been regression to the mean: the cases with the least and most reliable scores in wave 1 probably had moderately reliable scores in wave 2. Thus, those who appeared to have the potential to change may have simply had an artificially low starting score due to unreliability. Had there been three or more waves, we could have use latent growth curve analyses with a quadratic slope, a method with increased reliability.

Another weakness of the study is that there are some alternative explanations for the deleterious effects of excessive change. Because the data come from self-report instruments, excessive change may have been reported even when it did not occur. For instance, some participants may have lacked self-insight, or may have answered carelessly and hastily. However, our explanation for why excessive change is bad derives from perceived change rather than actual change. Given the satisfactory reliability of each scale, we also doubt that the typical participant answered carelessly.

## Conclusion

Research now shows the potential for change in personality traits through adulthood, and the benefits of positive trait change in terms of increased well-being. Our study adds to this growing literature by investigating whether and what levels of change and stability in personality traits are beneficial to well-being. We found evidence supporting the benefits of both stability and change in personality traits.

First, adults who remained at moderate or high trait levels (i.e., moderate and high sustainers) reported significantly higher levels of psychological well-being, higher positive affect, and lower negative affect than adults who remained at a lower trait levels (i.e., low sustainers). However, retaining the same high level of each trait produced more improvements in nearly all well-being outcomes than retaining the same level at a moderate level of each trait. Personality stability in adulthood is most conducive to improvements in well-being when individuals have achieved a sufficiently high level of extraversion (sociality and agency) and conscientiousness and a sufficiently low level of neuroticism. Our findings are in accord with the equanimity view of stability as epitomized by the Zen Buddhist maxim, “Cultivate and nourish yourself to enact maturity and achieve stability” [104].

Among adults with below average trait levels to start with, there was a “just right” moderate amount of increase in sociality, agency, and conscientiousness that produced the highest levels of PWB, and EWB; and the lowest level of NA. There was no diminishment of the benefits of declining neuroticism. It seems that one cannot experience too much change when it comes to neuroticism. In all, we found support for the Goldilocks hypothesis of positive trait change in nine of the twelve (i.e., 4 traits by 3 well-being outcomes) analyses.

## Supporting Information

S1 File Polynomial Regression Results. (DOCX)

S2 File Piecewise Regression Results. (DOCX)
