# Correction: Investigating the Goldilocks Hypothesis: The Non-Linear Impact of Positive Trait Change on Well-Being

**DOI:** 10.1371/journal.pone.0139760

**Published:** 2015-09-29

**Authors:** Chris C. Martin, Corey L. M. Keyes

There is an error in the fifth sentence of the second paragraph under the sub-subheading Subjective Well-Being, within the subheading Measures in the Methods section. This sentence should read as follows:

Life satisfaction was measured with a five-item scale in which participants rated their life overall, work situation, health, relationship with spouse (or partner), and relationship with children on a scale from 0 (worst possible) to 10 (best possible) [84]. The internal consistency of this scale was .67 in MIDUS I and .64 in MIDUS II (University of Wisconsin Institute on Aging, 2010 and 2009).

The references are: University of Wisconsin Institute on Aging. Documentation of Psychosocial Constructs and Composite Variables in MIDUS II Project 1. Ann Arbor, Michigan: Inter-University Consortium for Political and Social Research; 2010. Report No.: ICPSR 4652.

University of Wisconsin Institute on Aging. Documentation of Scales and Constructed Variables in MIDUS. Ann Arbor, Michigan: Inter-University Consortium for Political and Social Research; 2009. Report No.: ICPSR 2760.
