# Supplementary material for: Investigating the Goldilocks Hypothesis: The Non-Linear Impact of Positive Trait Change on Well-Being
Source: PLoS One. 2015 Jul 10;10(7):e0131316. doi: 10.1371/journal.pone.0131316 (PMC4498833; doi:10.1371/journal.pone.0131316)
Supplement: S1 File — (DOCX) [file pone.0131316.s001.docx]

**S1.Polynomial Regression Results**

Polynomial Regression Results for Sociality

|  | EWB 2 | |  | Absence of NA 2 | |  | PWB 2 | |
| --- | --- | --- | --- | --- | --- | --- | --- | --- |
|  | B | SE_B/_CI_95%_ |  | B | SE_B_ |  | B | SE_B_ |
| Constant | 4.98^***^ | 0.03 |  | 5.91^***^ | 0.04 |  | 5.49^***^ | 0.03 |
| Time-1 Value | 0.48^***^ | 0.02 |  | 0.46^***^ | 0.03 |  | 0.54^***^ | 0.02 |
| Age | 0.19^***^ | 0.04 |  | 0.13^*^ | 0.05 |  | 0.12^***^ | 0.03 |
| Age^2^ | -0.01 | 0.02 |  | -.01 | 0.02 |  | -0.20^***^ | 0.04 |
| Age^3^ | -0.06^***^ | 0.02 |  | -.04^†^ | 0.02 |  | -0.06^***^ | 0.01 |
| Age^4^ |  |  |  |  |  |  | 0.05^***^ | 0.01 |
| E1 | -0.37^***^ | 0.07 |  | -0.59^***^ | 0.10 |  | -0.29^***^ | 0.06 |
| E2 | 0.70^***^ | 0.07 |  | 0.66^***^ | 0.10 |  | 0.64^***^ | 0.06 |
| E1^2^ | -0.08 | 0.06 |  | 0.00 | 0.10 |  | -0.10 | 0.07 |
| E1 x E2 | 0.34^***^ | 0.10 |  | 0.40^*^ | 0.17 |  | 0.31^**^ | 0.12 |
| E2^2^ | -0.32^***^ | 0.07 |  | -0.36^***^ | 0.11 |  | -0.23^***^ | 0.07 |
| R^2^ | .42^***^ |  |  | .31^***^ |  |  | .47^***^ |  |

*Note*. Absence of NA was square-root transformed prior to analysis in order to minimize skewness. ^†^ p < .10, ^*^ p < .05, ^**^ p < .01, ^***^ p < .001.

Polynomial Regression Results for Agency

|  | EWB 2 | |  | Absence of NA 2 | |  | PWB 2 | |
| --- | --- | --- | --- | --- | --- | --- | --- | --- |
|  | B | SE_B_ |  | B | SE_B_ |  | B | SE_B_ |
| Constant | 5.11^***^ | 0.03 |  | 5.98^***^ | 0.04 |  | 5.62^***^ | 0.03 |
| Time-1 Value | 0.52^***^ | 0.02 |  | 0.46^***^ | 0.03 |  | 0.57^***^ | 0.02 |
| Age | 0.22^***^ | 0.04 |  | 0.14^*^ | 0.05 |  | 0.15^***^ | 0.03 |
| Age^2^ | -0.01 | 0.02 |  | -0.01 | 0.02 |  | -0.16^***^ | 0.05 |
| Age^3^ | -0.08^***^ | 0.02 |  | -0.05^*^ | 0.02 |  | -0.07^***^ | 0.01 |
| Age^4^ |  |  |  |  |  |  | 0.04^**^ | 0.01 |
| A1 | -0.01 | 0.06 |  | -0.18^***^ | 0.05 |  | -0.13^**^ | 0.04 |
| A2 | 0.16^*^ | 0.06 |  | 0.28^***^ | 0.05 |  | 0.38^***^ | 0.04 |
| A1^2^ | -0.06 | 0.05 |  | -0.01 | 0.08 |  | -0.02 | 0.06 |
| A1 x A2 | 0.25^**^ | 0.08 |  | 0.38^**^ | 0.14 |  | 0.14 | 0.10 |
| A2^2^ | -0.26^***^ | 0.06 |  | -0.43^***^ | 0.09 |  | -0.16^*^ | 0.07 |
| A1^3^ | -0.09 | 0.06 |  |  |  |  |  |  |
| A1^2 x^ A2 | 0.15 | 0.08 |  |  |  |  |  |  |
| A1 ^x^ A2^2^ | -0.22 | 0.08 |  |  |  |  |  |  |
| A3^3^ | 0.15 | 0.06 |  |  |  |  |  |  |
| R^2^ | .35^***^ |  |  | .28^***^ |  |  | .42^***^ |  |

*Note*. Absence of NA was square-root transformed prior to analysis in order to minimize skewness. ^*^ p < .05, ^**^ p < .01, ^***^ p < .001.

Polynomial Regression Results for Conscientiousness

|  | EWB 2 | |  | Absence of NA 2 | |  | PWB 2 | |
| --- | --- | --- | --- | --- | --- | --- | --- | --- |
|  | B | SE_B_ |  | B | SE_B_ |  | B | SE_B_ |
| Constant | 4.71^***^ | 0.06 |  | 5.51**^***^** | 0.10 |  | 5.14^***^ | 0.05 |
| Time-1 Value | 0.50^***^ | 0.02 |  | 0.45^***^ | 0.03 |  | 0.55^***^ | 0.02 |
| Age | 0.23^***^ | 0.04 |  | 0.16^***^ | 0.05 |  | 0.17^***^ | 0.03 |
| Age^2^ | 0.00 | 0.02 |  | -0.01 | 0.02 |  | -0.16^***^ | 0.04 |
| Age^3^ | -0.07^***^ | 0.02 |  | -0.05^*^ | 0.02 |  | -0.07^***^ | 0.01 |
| Age^4^ |  |  |  |  |  |  | 0.04^**^ | 0.01 |
| C1 | -0.11 | 0.10 |  | -0.27^*^ | 0.14 |  | -0.07 | 0.07 |
| C2 | 0.63^***^ | 0.13 |  | 0.89^***^ | 0.22 |  | 0.75^***^ | 0.10 |
| C1^2^ | -0.09 | 0.06 |  | 0.02 | 0.08 |  | -0.12^*^ | 0.05 |
| C1 x C2 | 0.16 | 0.11 |  | 0.22 | 0.17 |  | 0.14 | 0.09 |
| C2^2^ | -0.21^*^ | 0.09 |  | -0.43^**^ | 0.16 |  | -0.20^*^ | 0.08 |
| R^2^ | .35^***^ |  |  | .27^***^ |  |  | .42^***^ |  |

*Note*. Absence of NA was square-root transformed prior to analysis in order to minimize skewness., ^*^ p < .05, ^**^ p < .01, ^***^ p < .001.

*Polynomial Regression Results for Neuroticism (Reverse Scored)*

|  | EWB 2 | |  | Absence of NA 2 | |  | PWB 2 | |
| --- | --- | --- | --- | --- | --- | --- | --- | --- |
|  | B | SE_B_ |  | B | SE_B_ |  | B | SE_B_ |
| Constant | 4.90^***^ | .02 |  | 5.64^***^ | .04 |  | 5.48^***^ | .03 |
| Time-1 Value | 0.49^***^ | .03 |  | 0.34^***^ | .03 |  | 0.55^***^ | .02 |
| Age | 0.18^***^ | .06 |  | 0.07 | .04 |  | 0.12^***^ | .03 |
| Age^2^ | -0.01 | .02 |  | 0.02 | .02 |  | -0.18^***^ | .04 |
| Age^3^ | -0.07^***^ |  |  | -0.03 | .02 |  | -0.06^***^ | .01 |
| Age^4^ |  |  |  |  |  |  | 0.04^**^ | .01 |
| N1 (rev.) | -0.20^***^ | .03 |  | -0.19^**^ | .06 |  | -0.16^***^ | .03 |
| N2 (rev.) | 0.51^***^ | .04 |  | 0.87^***^ | .06 |  | 0.44^***^ | .04 |
| N1^2^ |  |  |  | 0.11^*^ | .05 |  |  |  |
| N1 x N2 |  |  |  | -0.07 | .09 |  |  |  |
| N2^2^ |  |  |  | -0.14^*^ | .07 |  |  |  |
| R^2^ | .41^***^ |  |  | .40^***^ |  |  | .43^***^ |  |

*Note*. Absence of NA was square-root transformed prior to analysis in order to minimize skewness. ^*^ p < .05, ^**^ p < .01, ^***^ p < .001.
