# Supplementary material for: Investigating the Goldilocks Hypothesis: The Non-Linear Impact of Positive Trait Change on Well-Being
Source: PLoS One. 2015 Jul 10;10(7):e0131316. doi: 10.1371/journal.pone.0131316 (PMC4498833; doi:10.1371/journal.pone.0131316)
Supplement: S2 File — (DOCX) [file pone.0131316.s002.docx]

**S1.Piecewise Regression Results**

The following tables contains the Stata output for the piecewise regression analyses, which were used to compute separate slopes for moderate changes and large changers. The tables are in pairs. Within each pair the first the first table contains three coefficients (e.g., Emoderate, Elarge, Einterval) that denote the slope for moderate changers, the slope for major changers, and the distance between the predicted score for a participants at 1 SD above the mean vs. 0.999 SD above the mean respectively. The second table in the pair has a coefficient (e.g. Elarge) that denotes the difference the first two coefficients in the previous table. If this distance is statistically significant, it indicates that the slope for moderate changers differs from the slope for large changers.

These coefficients of interest are in a bold font.

**PA and Sociality**

. regress M2ewbRescaled Emoderate Elarge Einterval m1ewb_mcenter ZageM1 ZageM1sq ZageM1cu

Source | SS df MS Number of obs = 1508

-------------+------------------------------ F( 7, 1500) = 139.47

Model | 371.231826 7 53.0331179 Prob > F = 0.0000

Residual | 570.370617 1500 .380247078 R-squared = 0.3943

-------------+------------------------------ Adj R-squared = 0.3914

Total | 941.602443 1507 .624819139 Root MSE = .61664

-------------------------------------------------------------------------------

M2ewbRescaled | Coef. Std. Err. t P>|t| [95% Conf. Interval]

--------------+----------------------------------------------------------------

**Emoderate | .2252651 .0222348 10.13 0.000 .1816504 .2688797**

**Elarge | .05869 .0782589 0.75 0.453 -.0948185 .2121985**

**Einterval | -.1240276 .0662376 -1.87 0.061 -.2539558 .0059006**

m1ewb_mcenter | .5520264 .0201495 27.40 0.000 .5125023 .5915505

ZageM1 | .2116287 .0357445 5.92 0.000 .1415142 .2817431

ZageM1sq | -.0147703 .0158231 -0.93 0.351 -.0458081 .0162674

ZageM1cu | -.0729229 .0154267 -4.73 0.000 -.1031831 -.0426627

_cons | 5.130868 .0239619 214.13 0.000 5.083865 5.17787

-------------------------------------------------------------------------------

. regress M2ewbRescaled EMmoderate EMlarge Einterval m1ewb_mcenter ZageM1 ZageM1sq ZageM1cu

Source | SS df MS Number of obs = 1508

-------------+------------------------------ F( 7, 1500) = 139.47

Model | 371.231826 7 53.0331179 Prob > F = 0.0000

Residual | 570.370617 1500 .380247078 R-squared = 0.3943

-------------+------------------------------ Adj R-squared = 0.3914

Total | 941.602443 1507 .624819139 Root MSE = .61664

-------------------------------------------------------------------------------

M2ewbRescaled | Coef. Std. Err. t P>|t| [95% Conf. Interval]

--------------+----------------------------------------------------------------

EMmoderate | .2252651 .0222348 10.13 0.000 .1816504 .2688797

**EMlarge | -.1665751 .08141 -2.05 0.041 -.3262646 -.0068856**

Einterval | -.1240276 .0662376 -1.87 0.061 -.2539558 .0059006

m1ewb_mcenter | .5520264 .0201495 27.40 0.000 .5125023 .5915505

ZageM1 | .2116287 .0357445 5.92 0.000 .1415142 .2817431

ZageM1sq | -.0147703 .0158231 -0.93 0.351 -.0458081 .0162674

ZageM1cu | -.0729229 .0154267 -4.73 0.000 -.1031831 -.0426627

_cons | 5.130868 .0239619 214.13 0.000 5.083865 5.17787

-------------------------------------------------------------------------------

---------------------------------------------------------------------------------------------------------------

**Absence of NA and Sociality**

. regress m2NAabsence Emoderate Elarge Einterval m1ewb_mcenter ZageM1 ZageM1sq ZageM1cu

Source | SS df MS Number of obs = 1508

-------------+------------------------------ F( 7, 1500) = 48.18

Model | 267.299815 7 38.1856878 Prob > F = 0.0000

Residual | 1188.9649 1500 .792643265 R-squared = 0.1836

-------------+------------------------------ Adj R-squared = 0.1797

Total | 1456.26471 1507 .966333585 Root MSE = .89031

-------------------------------------------------------------------------------

m2NAabsence | Coef. Std. Err. t P>|t| [95% Conf. Interval]

--------------+----------------------------------------------------------------

**Emoderate | .2287496 .0321026 7.13 0.000 .1657789 .2917203**

**Elarge | -.0138014 .1129899 -0.12 0.903 -.2354365 .2078336**

**Einterval | -.0736487 .0956336 -0.77 0.441 -.2612386 .1139412**

m1ewb_mcenter | .4518317 .0290917 15.53 0.000 .3947669 .5088964

ZageM1 | .1213891 .0516077 2.35 0.019 .0201581 .2226201

ZageM1sq | -.0568616 .0228453 -2.49 0.013 -.1016738 -.0120494

ZageM1cu | -.0270656 .022273 -1.22 0.224 -.0707552 .016624

_cons | 6.010972 .0345962 173.75 0.000 5.94311 6.078834

-------------------------------------------------------------------------------

. regress m2NAabsence EMmoderate EMlarge Einterval m1ewb_mcenter ZageM1 ZageM1sq ZageM1cu

Source | SS df MS Number of obs = 1508

-------------+------------------------------ F( 7, 1500) = 48.18

Model | 267.299815 7 38.1856878 Prob > F = 0.0000

Residual | 1188.9649 1500 .792643265 R-squared = 0.1836

-------------+------------------------------ Adj R-squared = 0.1797

Total | 1456.26471 1507 .966333585 Root MSE = .89031

-------------------------------------------------------------------------------

m2NAabsence | Coef. Std. Err. t P>|t| [95% Conf. Interval]

--------------+----------------------------------------------------------------

EMmoderate | .2287496 .0321026 7.13 0.000 .1657789 .2917203

**EMlarge | -.242551 .1175395 -2.06 0.039 -.4731102 -.0119919**

Einterval | -.0736487 .0956336 -0.77 0.441 -.2612386 .1139412

m1ewb_mcenter | .4518317 .0290917 15.53 0.000 .3947669 .5088964

ZageM1 | .1213891 .0516077 2.35 0.019 .0201581 .2226201

ZageM1sq | -.0568616 .0228453 -2.49 0.013 -.1016738 -.0120494

ZageM1cu | -.0270656 .022273 -1.22 0.224 -.0707552 .016624

_cons | 6.010972 .0345962 173.75 0.000 5.94311 6.078834

------------------------------------------------------------------------------- --------------------------------------------------------------------------------

**PWB and Sociality**

. regress M2pwbRescaled Emoderate Elarge Einterval m1ewb_mcenter ZageM1 ZageM1sq ZageM1cu ZageM1qu

Source | SS df MS Number of obs = 1508

-------------+------------------------------ F( 8, 1499) = 63.74

Model | 230.287473 8 28.7859342 Prob > F = 0.0000

Residual | 676.938254 1499 .451593232 R-squared = 0.2538

-------------+------------------------------ Adj R-squared = 0.2499

Total | 907.225728 1507 .602007782 Root MSE = .67201

-------------------------------------------------------------------------------

M2pwbRescaled | Coef. Std. Err. t P>|t| [95% Conf. Interval]

--------------+----------------------------------------------------------------

**Emoderate | .2167032 .0242484 8.94 0.000 .1691388 .2642677**

**Elarge | .0128314 .0853167 0.15 0.880 -.1545214 .1801842**

**Einterval | -.1080863 .0723409 -1.49 0.135 -.2499864 .0338138**

m1ewb_mcenter | .430292 .0219587 19.60 0.000 .387219 .4733651

ZageM1 | .102745 .0392979 2.61 0.009 .0256604 .1798296

ZageM1sq | -.1793793 .0538104 -3.33 0.001 -.2849311 -.0738276

ZageM1cu | -.0597967 .0173489 -3.45 0.001 -.0938274 -.025766

ZageM1qu | .0359549 .0166887 2.15 0.031 .0032192 .0686905

_cons | 5.707429 .031164 183.14 0.000 5.6463 5.768559

-------------------------------------------------------------------------------

. regress M2pwbRescaled EMmoderate EMlarge Einterval m1ewb_mcenter ZageM1 ZageM1sq ZageM1cu ZageM1qu

Source | SS df MS Number of obs = 1508

-------------+------------------------------ F( 8, 1499) = 63.74

Model | 230.287473 8 28.7859342 Prob > F = 0.0000

Residual | 676.938254 1499 .451593232 R-squared = 0.2538

-------------+------------------------------ Adj R-squared = 0.2499

Total | 907.225728 1507 .602007782 Root MSE = .67201

-------------------------------------------------------------------------------

M2pwbRescaled | Coef. Std. Err. t P>|t| [95% Conf. Interval]

--------------+----------------------------------------------------------------

EMmoderate | .2167032 .0242484 8.94 0.000 .1691388 .2642677

**EMlarge | -.2038718 .0887304 -2.30 0.022 -.3779207 -.0298229**

Einterval | -.1080863 .0723409 -1.49 0.135 -.2499864 .0338138

m1ewb_mcenter | .430292 .0219587 19.60 0.000 .387219 .4733651

ZageM1 | .102745 .0392979 2.61 0.009 .0256604 .1798296

ZageM1sq | -.1793793 .0538104 -3.33 0.001 -.2849311 -.0738276

ZageM1cu | -.0597967 .0173489 -3.45 0.001 -.0938274 -.025766

ZageM1qu | .0359549 .0166887 2.15 0.031 .0032192 .0686905

_cons | 5.707429 .031164 183.14 0.000 5.6463 5.768559

-------------------------------------------------------------------------------

---------------------------------------------------------------------------------------------------------------

**PA and Agency**

. regress M2ewbRescaled Amoderate Alarge Ainterval m1ewb_mcenter ZageM1 ZageM1sq ZageM1cu

Source | SS df MS Number of obs = 1508

-------------+------------------------------ F( 7, 1500) = 118.62

Model | 335.506114 7 47.9294448 Prob > F = 0.0000

Residual | 606.096329 1500 .404064219 R-squared = 0.3563

-------------+------------------------------ Adj R-squared = 0.3533

Total | 941.602443 1507 .624819139 Root MSE = .63566

-------------------------------------------------------------------------------

M2ewbRescaled | Coef. Std. Err. t P>|t| [95% Conf. Interval]

--------------+----------------------------------------------------------------

**Amoderate | .1090629 .0245383 4.44 0.000 .0609299 .1571959**

**Alarge | -.0096187 .077787 -0.12 0.902 -.1622015 .1429641**

**Ainterval | -.0294469 .0607878 -0.48 0.628 -.148685 .0897912**

m1ewb_mcenter | .5494301 .0207362 26.50 0.000 .5087552 .590105

ZageM1 | .2375639 .0366222 6.49 0.000 .1657278 .3094

ZageM1sq | -.0172454 .016321 -1.06 0.291 -.0492598 .014769

ZageM1cu | -.0852699 .0158317 -5.39 0.000 -.1163244 -.0542153

_cons | 5.116292 .0258986 197.55 0.000 5.065491 5.167094

-------------------------------------------------------------------------------

. regress M2ewbRescaled AMmoderate AMlarge Ainterval m1ewb_mcenter ZageM1 ZageM1sq ZageM1cu

Source | SS df MS Number of obs = 1508

-------------+------------------------------ F( 7, 1500) = 118.62

Model | 335.506114 7 47.9294448 Prob > F = 0.0000

Residual | 606.096329 1500 .404064219 R-squared = 0.3563

-------------+------------------------------ Adj R-squared = 0.3533

Total | 941.602443 1507 .624819139 Root MSE = .63566

-------------------------------------------------------------------------------

M2ewbRescaled | Coef. Std. Err. t P>|t| [95% Conf. Interval]

--------------+----------------------------------------------------------------

AMmoderate | .1090629 .0245383 4.44 0.000 .0609299 .1571959

**AMlarge | -.1186816 .0816013 -1.45 0.146 -.2787465 .0413832**

Ainterval | -.0294469 .0607878 -0.48 0.628 -.148685 .0897912

m1ewb_mcenter | .5494301 .0207362 26.50 0.000 .5087552 .590105

ZageM1 | .2375639 .0366222 6.49 0.000 .1657278 .3094

ZageM1sq | -.0172454 .016321 -1.06 0.291 -.0492598 .014769

ZageM1cu | -.0852699 .0158317 -5.39 0.000 -.1163244 -.0542153

_cons | 5.116292 .0258986 197.55 0.000 5.065491 5.167094

-------------------------------------------------------------------------------

---------------------------------------------------------------------------------------------------------------

**Absence of NA and Agency**

. regress m2NAabsence Amoderate Alarge Ainterval m1ewb_mcenter ZageM1 ZageM1sq ZageM1cu

Source | SS df MS Number of obs = 1508

-------------+------------------------------ F( 7, 1500) = 40.91

Model | 233.457444 7 33.3510635 Prob > F = 0.0000

Residual | 1222.80727 1500 .815204846 R-squared = 0.1603

-------------+------------------------------ Adj R-squared = 0.1564

Total | 1456.26471 1507 .966333585 Root MSE = .90289

-------------------------------------------------------------------------------

m2NAabsence | Coef. Std. Err. t P>|t| [95% Conf. Interval]

--------------+----------------------------------------------------------------

**Amoderate | .131615 .034854 3.78 0.000 .0632472 .1999827**

**Alarge | -.0773651 .110488 -0.70 0.484 -.2940924 .1393623**

**Ainterval | -.0294059 .0863425 -0.34 0.733 -.1987707 .1399589**

m1ewb_mcenter | .4486218 .0294535 15.23 0.000 .3908474 .5063961

ZageM1 | .149723 .0520178 2.88 0.004 .0476876 .2517584

ZageM1sq | -.0583273 .0231822 -2.52 0.012 -.1038003 -.0128543

ZageM1cu | -.0406867 .0224872 -1.81 0.071 -.0847963 .003423

_cons | 6.004641 .0367861 163.23 0.000 5.932483 6.076799

-------------------------------------------------------------------------------

. regress m2NAabsence AMmoderate AMlarge Ainterval m1ewb_mcenter ZageM1 ZageM1sq ZageM1cu

Source | SS df MS Number of obs = 1508

-------------+------------------------------ F( 7, 1500) = 40.91

Model | 233.457444 7 33.3510635 Prob > F = 0.0000

Residual | 1222.80727 1500 .815204846 R-squared = 0.1603

-------------+------------------------------ Adj R-squared = 0.1564

Total | 1456.26471 1507 .966333585 Root MSE = .90289

-------------------------------------------------------------------------------

m2NAabsence | Coef. Std. Err. t P>|t| [95% Conf. Interval]

--------------+----------------------------------------------------------------

AMmoderate | .131615 .034854 3.78 0.000 .0632472 .1999827

**AMlarge | -.2089801 .1159059 -1.80 0.072 -.4363349 .0183748**

Ainterval | -.0294059 .0863425 -0.34 0.733 -.1987707 .1399589

m1ewb_mcenter | .4486218 .0294535 15.23 0.000 .3908474 .5063961

ZageM1 | .149723 .0520178 2.88 0.004 .0476876 .2517584

ZageM1sq | -.0583273 .0231822 -2.52 0.012 -.1038003 -.0128543

ZageM1cu | -.0406867 .0224872 -1.81 0.071 -.0847963 .003423

_cons | 6.004641 .0367861 163.23 0.000 5.932483 6.076799

-------------------------------------------------------------------------------

---------------------------------------------------------------------------------------------------------------

**PWB and Agency**

. regress M2pwbRescaled Amoderate Alarge Ainterval m1ewb_mcenter ZageM1 ZageM1sq ZageM1cu ZageM1qu

Source | SS df MS Number of obs = 1508

-------------+------------------------------ F( 8, 1499) = 53.37

Model | 201.123993 8 25.1404992 Prob > F = 0.0000

Residual | 706.101734 1499 .471048522 R-squared = 0.2217

-------------+------------------------------ Adj R-squared = 0.2175

Total | 907.225728 1507 .602007782 Root MSE = .68633

-------------------------------------------------------------------------------

M2pwbRescaled | Coef. Std. Err. t P>|t| [95% Conf. Interval]

--------------+----------------------------------------------------------------

**Amoderate | .1199842 .0265545 4.52 0.000 .0678963 .172072**

**Alarge | .0427112 .0839955 0.51 0.611 -.12205 .2074723**

**Ainterval | -.0605033 .0656376 -0.92 0.357 -.1892547 .0682481**

m1ewb_mcenter | .4285584 .0223894 19.14 0.000 .3846405 .4724763

ZageM1 | .1254071 .0398858 3.14 0.002 .0471692 .2036451

ZageM1sq | -.153875 .0550356 -2.80 0.005 -.26183 -.0459199

ZageM1cu | -.0697539 .0176344 -3.96 0.000 -.1043446 -.0351633

ZageM1qu | .0270087 .0170517 1.58 0.113 -.0064389 .0604564

_cons | 5.685893 .0324012 175.48 0.000 5.622336 5.749449

-------------------------------------------------------------------------------

. regress M2pwbRescaled AMmoderate AMlarge Ainterval m1ewb_mcenter ZageM1 ZageM1sq ZageM1cu ZageM1qu

Source | SS df MS Number of obs = 1508

-------------+------------------------------ F( 8, 1499) = 53.37

Model | 201.123993 8 25.1404992 Prob > F = 0.0000

Residual | 706.101734 1499 .471048522 R-squared = 0.2217

-------------+------------------------------ Adj R-squared = 0.2175

Total | 907.225728 1507 .602007782 Root MSE = .68633

-------------------------------------------------------------------------------

M2pwbRescaled | Coef. Std. Err. t P>|t| [95% Conf. Interval]

--------------+----------------------------------------------------------------

AMmoderate | .1199842 .0265545 4.52 0.000 .0678963 .172072

**AMlarge | -.077273 .0881552 -0.88 0.381 -.2501936 .0956476**

Ainterval | -.0605033 .0656376 -0.92 0.357 -.1892547 .0682481

m1ewb_mcenter | .4285584 .0223894 19.14 0.000 .3846405 .4724763

ZageM1 | .1254071 .0398858 3.14 0.002 .0471692 .2036451

ZageM1sq | -.153875 .0550356 -2.80 0.005 -.26183 -.0459199

ZageM1cu | -.0697539 .0176344 -3.96 0.000 -.1043446 -.0351633

ZageM1qu | .0270087 .0170517 1.58 0.113 -.0064389 .0604564

_cons | 5.685893 .0324012 175.48 0.000 5.622336 5.749449

-------------------------------------------------------------------------------

**PA and Conscientiousness**

. regress M2ewbRescaled Cmoderate Clarge Cinterval m1ewb_mcenter ZageM1 ZageM1sq ZageM1cu

Source | SS df MS Number of obs = 1508

-------------+------------------------------ F( 7, 1500) = 115.70

Model | 330.136814 7 47.162402 Prob > F = 0.0000

Residual | 611.465629 1500 .407643753 R-squared = 0.3506

-------------+------------------------------ Adj R-squared = 0.3476

Total | 941.602443 1507 .624819139 Root MSE = .63847

-------------------------------------------------------------------------------

M2ewbRescaled | Coef. Std. Err. t P>|t| [95% Conf. Interval]

--------------+----------------------------------------------------------------

**Cmoderate | .0783145 .0236813 3.31 0.001 .0318624 .1247665**

**Clarge | -.0530331 .0798801 -0.66 0.507 -.2097217 .1036554**

**Cinterval | .0206297 .0797023 0.26 0.796 -.13571 .1769695**

m1ewb_mcenter | .549299 .0209778 26.18 0.000 .5081501 .5904478

ZageM1 | .239124 .0367855 6.50 0.000 .1669675 .3112805

ZageM1sq | -.0211514 .0163669 -1.29 0.196 -.0532559 .0109531

ZageM1cu | -.0824688 .0159194 -5.18 0.000 -.1136955 -.0512421

_cons | 5.114296 .0243359 210.15 0.000 5.06656 5.162032

-------------------------------------------------------------------------------

. regress M2ewbRescaled CMmoderate CMlarge Cinterval m1ewb_mcenter ZageM1 ZageM1sq ZageM1cu

Source | SS df MS Number of obs = 1508

-------------+------------------------------ F( 7, 1500) = 115.70

Model | 330.136814 7 47.162402 Prob > F = 0.0000

Residual | 611.465629 1500 .407643753 R-squared = 0.3506

-------------+------------------------------ Adj R-squared = 0.3476

Total | 941.602443 1507 .624819139 Root MSE = .63847

-------------------------------------------------------------------------------

M2ewbRescaled | Coef. Std. Err. t P>|t| [95% Conf. Interval]

--------------+----------------------------------------------------------------

CMmoderate | .0783145 .0236813 3.31 0.001 .0318624 .1247665

**CMlarge | -.1313476 .0832851 -1.58 0.115 -.2947153 .0320201**

Cinterval | .0206297 .0797023 0.26 0.796 -.13571 .1769695

m1ewb_mcenter | .549299 .0209778 26.18 0.000 .5081501 .5904478

ZageM1 | .239124 .0367855 6.50 0.000 .1669675 .3112805

ZageM1sq | -.0211514 .0163669 -1.29 0.196 -.0532559 .0109531

ZageM1cu | -.0824688 .0159194 -5.18 0.000 -.1136955 -.0512421

_cons | 5.114296 .0243359 210.15 0.000 5.06656 5.162032

-------------------------------------------------------------------------------

**Absence of NA and Conscientiousness**

. regress m2NAabsence Cmoderate Clarge Cinterval m1ewb_mcenter ZageM1 ZageM1sq ZageM1cu

Source | SS df MS Number of obs = 1508

-------------+------------------------------ F( 7, 1500) = 41.49

Model | 236.21067 7 33.7443815 Prob > F = 0.0000

Residual | 1220.05404 1500 .813369362 R-squared = 0.1622

-------------+------------------------------ Adj R-squared = 0.1583

Total | 1456.26471 1507 .966333585 Root MSE = .90187

-------------------------------------------------------------------------------

m2NAabsence | Coef. Std. Err. t P>|t| [95% Conf. Interval]

--------------+----------------------------------------------------------------

**Cmoderate | .1294389 .0334511 3.87 0.000 .0638231 .1950548**

**Clarge | .101305 .1128345 0.90 0.369 -.1200252 .3226352**

**Cinterval | -.0621074 .1125834 -0.55 0.581 -.2829449 .1587302**

m1ewb_mcenter | .4542759 .0296321 15.33 0.000 .3961511 .5124007

ZageM1 | .1504611 .0519614 2.90 0.004 .0485364 .2523857

ZageM1sq | -.0628517 .0231191 -2.72 0.007 -.1082008 -.0175025

ZageM1cu | -.0357663 .022487 -1.59 0.112 -.0798755 .008343

_cons | 5.997005 .0343756 174.46 0.000 5.929575 6.064434

-------------------------------------------------------------------------------

. regress m2NAabsence CMmoderate CMlarge Cinterval m1ewb_mcenter ZageM1 ZageM1sq ZageM1cu

Source | SS df MS Number of obs = 1508

-------------+------------------------------ F( 7, 1500) = 41.49

Model | 236.21067 7 33.7443815 Prob > F = 0.0000

Residual | 1220.05404 1500 .813369362 R-squared = 0.1622

-------------+------------------------------ Adj R-squared = 0.1583

Total | 1456.26471 1507 .966333585 Root MSE = .90187

-------------------------------------------------------------------------------

m2NAabsence | Coef. Std. Err. t P>|t| [95% Conf. Interval]

--------------+----------------------------------------------------------------

CMmoderate | .1294389 .0334511 3.87 0.000 .0638231 .1950548

**CMlarge | -.0281339 .1176443 -0.24 0.811 -.2588988 .202631**

Cinterval | -.0621074 .1125834 -0.55 0.581 -.2829449 .1587302

m1ewb_mcenter | .4542759 .0296321 15.33 0.000 .3961511 .5124007

ZageM1 | .1504611 .0519614 2.90 0.004 .0485364 .2523857

ZageM1sq | -.0628517 .0231191 -2.72 0.007 -.1082008 -.0175025

ZageM1cu | -.0357663 .022487 -1.59 0.112 -.0798755 .008343

_cons | 5.997005 .0343756 174.46 0.000 5.929575 6.064434

-------------------------------------------------------------------------------

**PWB and Conscientiousness**

. regress M2pwbRescaled Cmoderate Clarge Cinterval m1ewb_mcenter ZageM1 ZageM1sq ZageM1cu ZageM1qu

Source | SS df MS Number of obs = 1508

-------------+------------------------------ F( 8, 1499) = 51.18

Model | 194.649212 8 24.3311515 Prob > F = 0.0000

Residual | 712.576515 1499 .475367922 R-squared = 0.2146

-------------+------------------------------ Adj R-squared = 0.2104

Total | 907.225728 1507 .602007782 Root MSE = .68947

-------------------------------------------------------------------------------

M2pwbRescaled | Coef. Std. Err. t P>|t| [95% Conf. Interval]

--------------+----------------------------------------------------------------

**Cmoderate | .0784115 .0255768 3.07 0.002 .0282414 .1285816**

**Clarge | -.188196 .0863009 -2.18 0.029 -.3574792 -.0189127**

**Cinterval | .0454084 .0861054 0.53 0.598 -.1234915 .2143082**

m1ewb_mcenter | .4230826 .0226541 18.68 0.000 .3786455 .4675196

ZageM1 | .12863 .0400565 3.21 0.001 .0500573 .2072028

ZageM1sq | -.176047 .0551099 -3.19 0.001 -.2841478 -.0679463

ZageM1cu | -.0688717 .0177279 -3.88 0.000 -.1036459 -.0340975

ZageM1qu | .0331208 .0170932 1.94 0.053 -.0004084 .0666499

_cons | 5.69648 .0312308 182.40 0.000 5.635219 5.75774

-------------------------------------------------------------------------------

. regress M2pwbRescaled CMmoderate CMlarge Cinterval m1ewb_mcenter ZageM1 ZageM1sq ZageM1cu ZageM1qu

Source | SS df MS Number of obs = 1508

-------------+------------------------------ F( 8, 1499) = 51.18

Model | 194.649212 8 24.3311515 Prob > F = 0.0000

Residual | 712.576515 1499 .475367922 R-squared = 0.2146

-------------+------------------------------ Adj R-squared = 0.2104

Total | 907.225728 1507 .602007782 Root MSE = .68947

-------------------------------------------------------------------------------

M2pwbRescaled | Coef. Std. Err. t P>|t| [95% Conf. Interval]

--------------+----------------------------------------------------------------

CMmoderate | .0784115 .0255768 3.07 0.002 .0282414 .1285816

**CMlarge | -.2666075 .0899644 -2.96 0.003 -.4430769 -.090138**

Cinterval | .0454084 .0861054 0.53 0.598 -.1234915 .2143082

m1ewb_mcenter | .4230826 .0226541 18.68 0.000 .3786455 .4675196

ZageM1 | .12863 .0400565 3.21 0.001 .0500573 .2072028

ZageM1sq | -.176047 .0551099 -3.19 0.001 -.2841478 -.0679463

ZageM1cu | -.0688717 .0177279 -3.88 0.000 -.1036459 -.0340975

ZageM1qu | .0331208 .0170932 1.94 0.053 -.0004084 .0666499

_cons | 5.69648 .0312308 182.40 0.000 5.635219 5.75774

-------------------------------------------------------------------------------.

**PA and Neuroticism (Reversed)**

. regress M2ewbRescaled Nmoderate Nlarge Ninterval m1ewb_mcenter ZageM1 ZageM1sq ZageM1cu

Source | SS df MS Number of obs = 1508

-------------+------------------------------ F( 7, 1500) = 134.03

Model | 362.320529 7 51.7600756 Prob > F = 0.0000

Residual | 579.281914 1500 .386187942 R-squared = 0.3848

-------------+------------------------------ Adj R-squared = 0.3819

Total | 941.602443 1507 .624819139 Root MSE = .62144

-------------------------------------------------------------------------------

M2ewbRescaled | Coef. Std. Err. t P>|t| [95% Conf. Interval]

--------------+----------------------------------------------------------------

**Nmoderate | .1843106 .0243822 7.56 0.000 .1364839 .2321374**

**Nlarge | .2128835 .067576 3.15 0.002 .0803301 .3454369**

**Ninterval | -.117697 .0664353 -1.77 0.077 -.2480128 .0126189**

m1ewb_mcenter | .5821052 .0206397 28.20 0.000 .5416194 .6225909

ZageM1 | .2275741 .0358334 6.35 0.000 .1572852 .297863

ZageM1sq | -.0225813 .0159392 -1.42 0.157 -.0538468 .0086843

ZageM1cu | -.079059 .0154886 -5.10 0.000 -.1094406 -.0486774

_cons | 5.121182 .0242907 210.83 0.000 5.073534 5.168829

-------------------------------------------------------------------------------

. regress M2ewbRescaled NMmoderate NMlarge Ninterval m1ewb_mcenter ZageM1 ZageM1sq ZageM1cu

Source | SS df MS Number of obs = 1508

-------------+------------------------------ F( 7, 1500) = 134.03

Model | 362.320529 7 51.7600756 Prob > F = 0.0000

Residual | 579.281914 1500 .386187942 R-squared = 0.3848

-------------+------------------------------ Adj R-squared = 0.3819

Total | 941.602443 1507 .624819139 Root MSE = .62144

-------------------------------------------------------------------------------

M2ewbRescaled | Coef. Std. Err. t P>|t| [95% Conf. Interval]

--------------+----------------------------------------------------------------

NMmoderate | .1843106 .0243822 7.56 0.000 .1364839 .2321374

**NMlarge | .0285729 .0717852 0.40 0.691 -.1122372 .169383**

Ninterval | -.117697 .0664353 -1.77 0.077 -.2480128 .0126189

m1ewb_mcenter | .5821052 .0206397 28.20 0.000 .5416194 .6225909

ZageM1 | .2275741 .0358334 6.35 0.000 .1572852 .297863

ZageM1sq | -.0225813 .0159392 -1.42 0.157 -.0538468 .0086843

ZageM1cu | -.079059 .0154886 -5.10 0.000 -.1094406 -.0486774

_cons | 5.121182 .0242907 210.83 0.000 5.073534 5.168829

-------------------------------------------------------------------------------

**Absence of NA and Neuroticism (Reversed)**

. regress m2NAabsence Nmoderate Nlarge Ninterval m1ewb_mcenter ZageM1 ZageM1sq ZageM1cu

Source | SS df MS Number of obs = 1508

-------------+------------------------------ F( 7, 1500) = 54.05

Model | 293.309097 7 41.9012996 Prob > F = 0.0000

Residual | 1162.95562 1500 .775303744 R-squared = 0.2014

-------------+------------------------------ Adj R-squared = 0.1977

Total | 1456.26471 1507 .966333585 Root MSE = .88051

-------------------------------------------------------------------------------

m2NAabsence | Coef. Std. Err. t P>|t| [95% Conf. Interval]

--------------+----------------------------------------------------------------

**Nmoderate | .3019913 .0345469 8.74 0.000 .234226 .3697566**

**Nlarge | .1800891 .0957478 1.88 0.060 -.0077247 .3679029**

**Ninterval | -.2392632 .0941316 -2.54 0.011 -.4239066 -.0546197**

m1ewb_mcenter | .4902705 .0292442 16.76 0.000 .4329066 .5476344

ZageM1 | .132528 .050772 2.61 0.009 .0329363 .2321197

ZageM1sq | -.0627054 .0225842 -2.78 0.006 -.1070053 -.0184055

ZageM1cu | -.0318747 .0219456 -1.45 0.147 -.0749221 .0111728

_cons | 6.035936 .0344173 175.37 0.000 5.968425 6.103447

-------------------------------------------------------------------------------

. regress m2NAabsence NMmoderate NMlarge Ninterval m1ewb_mcenter ZageM1 ZageM1sq ZageM1cu

Source | SS df MS Number of obs = 1508

-------------+------------------------------ F( 7, 1500) = 54.05

Model | 293.309097 7 41.9012996 Prob > F = 0.0000

Residual | 1162.95562 1500 .775303744 R-squared = 0.2014

-------------+------------------------------ Adj R-squared = 0.1977

Total | 1456.26471 1507 .966333585 Root MSE = .88051

-------------------------------------------------------------------------------

m2NAabsence | Coef. Std. Err. t P>|t| [95% Conf. Interval]

--------------+----------------------------------------------------------------

NMmoderate | .3019913 .0345469 8.74 0.000 .234226 .3697566

**NMlarge | -.1219022 .1017119 -1.20 0.231 -.3214148 .0776104**

Ninterval | -.2392632 .0941316 -2.54 0.011 -.4239066 -.0546197

m1ewb_mcenter | .4902705 .0292442 16.76 0.000 .4329066 .5476344

ZageM1 | .132528 .050772 2.61 0.009 .0329363 .2321197

ZageM1sq | -.0627054 .0225842 -2.78 0.006 -.1070053 -.0184055

ZageM1cu | -.0318747 .0219456 -1.45 0.147 -.0749221 .0111728

_cons | 6.035936 .0344173 175.37 0.000 5.968425 6.103447

-------------------------------------------------------------------------------

---------------------------------------------------------------------------------------------------------------

**PWB and Neuroticism (Reversed)**

. regress M2pwbRescaled Nmoderate Nlarge Ninterval m1ewb_mcenter ZageM1 ZageM1sq ZageM1cu ZageM1qu

Source | SS df MS Number of obs = 1508

-------------+------------------------------ F( 8, 1499) = 56.56

Model | 210.340705 8 26.2925881 Prob > F = 0.0000

Residual | 696.885023 1499 .464899949 R-squared = 0.2319

-------------+------------------------------ Adj R-squared = 0.2278

Total | 907.225728 1507 .602007782 Root MSE = .68184

-------------------------------------------------------------------------------

M2pwbRescaled | Coef. Std. Err. t P>|t| [95% Conf. Interval]

--------------+----------------------------------------------------------------

**Nmoderate | .1526872 .0267533 5.71 0.000 .1002093 .205165**

**Nlarge | .0968282 .0741794 1.31 0.192 -.0486782 .2423347**

**Ninterval | -.0750265 .0729086 -1.03 0.304 -.2180402 .0679873**

m1ewb_mcenter | .4518248 .0226467 19.95 0.000 .4074023 .4962474

ZageM1 | .1201952 .0396464 3.03 0.002 .042427 .1979635

ZageM1sq | -.1831796 .0545275 -3.36 0.001 -.2901379 -.0762214

ZageM1cu | -.0666985 .0175284 -3.81 0.000 -.1010814 -.0323157

ZageM1qu | .0352463 .0169028 2.09 0.037 .0020908 .0684019

_cons | 5.69614 .0314216 181.28 0.000 5.634505 5.757775

-------------------------------------------------------------------------------

. regress M2pwbRescaled NMmoderate NMlarge Ninterval m1ewb_mcenter ZageM1 ZageM1sq ZageM1cu ZageM1qu

Source | SS df MS Number of obs = 1508

-------------+------------------------------ F( 8, 1499) = 56.56

Model | 210.340705 8 26.2925881 Prob > F = 0.0000

Residual | 696.885023 1499 .464899949 R-squared = 0.2319

-------------+------------------------------ Adj R-squared = 0.2278

Total | 907.225728 1507 .602007782 Root MSE = .68184

-------------------------------------------------------------------------------

M2pwbRescaled | Coef. Std. Err. t P>|t| [95% Conf. Interval]

--------------+----------------------------------------------------------------

NMmoderate | .1526872 .0267533 5.71 0.000 .1002093 .205165

**NMlarge | -.0558589 .0787879 -0.71 0.478 -.2104051 .0986872**

Ninterval | -.0750265 .0729086 -1.03 0.304 -.2180402 .0679873

m1ewb_mcenter | .4518248 .0226467 19.95 0.000 .4074023 .4962474

ZageM1 | .1201952 .0396464 3.03 0.002 .042427 .1979635

ZageM1sq | -.1831796 .0545275 -3.36 0.001 -.2901379 -.0762214

ZageM1cu | -.0666985 .0175284 -3.81 0.000 -.1010814 -.0323157

ZageM1qu | .0352463 .0169028 2.09 0.037 .0020908 .0684019

_cons | 5.69614 .0314216 181.28 0.000 5.634505 5.757775

-------------------------------------------------------------------------------
